# Tumor cell-released kynurenine biases MEP differentiation into megakaryocytes in individuals with cancer by activating AhR–RUNX1

**DOI:** 10.1038/s41590-023-01662-3

**Published:** 2023-11-02

**Authors:** Li Zhou, Dongxiao Wu, Yabo Zhou, Dianheng Wang, Haixia Fu, Qiusha Huang, Guohui Qin, Jie Chen, Jiadi Lv, Shaoyang Lai, Huafeng Zhang, Ke Tang, Jingwei Ma, Roland Fiskesund, Yi Zhang, Xiaohui Zhang, Bo Huang

**Affiliations:** 1 Department of Immunology & National Key Laboratory of Medical Molecular Biology, Institute of Basic Medical Sciences, Chinese Academy of Medical Sciences (CAMS) & Peking Union Medical College, Beijing, China; 2 Peking University People’s Hospital, Peking University Institute of Hematology, National Clinical Research Center for Hematologic Disease, Beijing Key Laboratory of Hematopoietic Stem Cell Transplantation, Beijing, China; 3 Biotherapy Center and Cancer Center, The First Affiliated Hospital of Zhengzhou University, Zhengzhou, China; 4 The Department of Obstetrics, Women and Children’s Hospital, School of Medicine, Xiamen University, Xiamen, China; 5 Department of Pathology, School of Basic Medicine, Tongji Medical College, Huazhong University of Science and Technology, Wuhan, China; 6 Department of Biochemistry and Molecular Biology, Tongji Medical College, Huazhong University of Science and Technology, Wuhan, China; 7 Department of Immunology, School of Basic Medicine, Tongji Medical College, Huazhong University of Science and Technology, Wuhan, China; 8 Department of Clinical Immunology and Transfusion Medicine, Karolinska University Hospital, Stockholm, Sweden; 9 Department of Medicine, Karolinska Institutet, Huddinge, Sweden

**Keywords:** Haematopoietic stem cells, Haematopoietic stem cells

## Abstract

Tumor-derived factors are thought to regulate thrombocytosis and erythrocytopenia in individuals with cancer; however, such factors have not yet been identified. Here we show that tumor cell-released kynurenine (Kyn) biases megakaryocytic–erythroid progenitor cell (MEP) differentiation into megakaryocytes in individuals with cancer by activating the aryl hydrocarbon receptor–Runt-related transcription factor 1 (AhR–RUNX1) axis. During tumor growth, large amounts of Kyn from tumor cells are released into the periphery, where they are taken up by MEPs via the transporter SLC7A8. In the cytosol, Kyn binds to and activates AhR, leading to its translocation into the nucleus where AhR transactivates RUNX1, thus regulating MEP differentiation into megakaryocytes. In addition, activated AhR upregulates SLC7A8 in MEPs to induce positive feedback. Importantly, Kyn–AhR–RUNX1-regulated MEP differentiation was demonstrated in both humanized mice and individuals with cancer, providing potential strategies for the prevention of thrombocytosis and erythrocytopenia.

## Abstract (web summary)

Individuals with advanced cancers can develop thrombocytosis and anemia. Huang and colleagues show that in tumor-bearing individuals, increased circulating kynurenine results in megakaryocyte differentiation from megakaryocytic–erythroid progenitor cells by activating the aryl hydrocarbon receptor, resulting in increased expression of RUNX1.

## Main

Thrombocytosis and erythrocytopenia are common consequences of abnormal hematopoiesis in individuals with advanced cancer^1,2^. Enhanced megakaryopoiesis increases platelet (PLT) numbers, which potentially induces thrombogenesis and tumor cell metastasis in individuals with cancer^3–5^. Simultaneously, erythrocytopenia occurs, which causes anemia and exacerbates tumor hypoxia^2,6^. Both anemia and thrombogenesis mark poor prognosis in various types of cancers^2,7,8^. Despite the important roles of erythrocytopenia and thrombocytosis in tumor progression, metastasis and treatment resistance, the molecular mechanisms of erythrocytopenia and thrombocytosis formation remain largely unclear. Notably, PLTs and erythrocytes are differentiated from the common megakaryocytic–erythroid progenitor cells (MEPs)^9,10^. Many transcription factors, such as GATA binding protein 1 (GATA1), GATA2, Runt-related transcription factor 1 (RUNX1), T cell acute lymphocytic leukemia 1 (TAL1), friend leukemia integration 1 (FLI1), MYB and Krueppel-like factor 1 (KLF1), are involved in megakaryocytic–erythroid differentiation^11–14^. RUNX1 and GATA1 are pivotal for megakaryocytic and erythroid differentiation, respectively^15^. Despite physiological regulation, little is known about how tumor-derived factors disrupt normal MEP differentiation and cause erythrocytopenia and thrombocytosis.

Aryl hydrocarbon receptor (AhR), a member of the basic helix-loop-helix family of transcription factors, plays an essential role in a wide variety of cells, including hematopoietic progenitor cells^16,17^. Notably, defects in PLT number and function have been observed in *Ahr*-null mice^18^; however, the AhR inhibitor stemregenin 1 (SR1) may promote erythropoiesis from human embryonic stem cells^19^, suggesting that AhR possibly regulates megakaryocytic–erythroid differentiation. As a cytoplasmic transcription factor, AhR can be activated by exogenous xenobiotics, such as 2,3,7,8-tetrachlorodibenzo-*p*-dioxin, and endogenous metabolites, including kynurenine (Kyn)^20,21^. Our previous studies have demonstrated that both tumor cells and immune cells mobilize the tryptophan–Kyn–AhR circuitry to dampen antitumor immunity^22,23^, and high levels of Kyn are present in the peripheral blood of individuals with cancer^23,24^. Based on these lines of information, we hypothesized that in individuals with cancer, Kyn is mobilized to trigger thrombocytosis and erythrocytopenia by activating AhR.

## Results

### Abnormal MEP differentiation occurs in advanced tumors

**Fig. 1:**
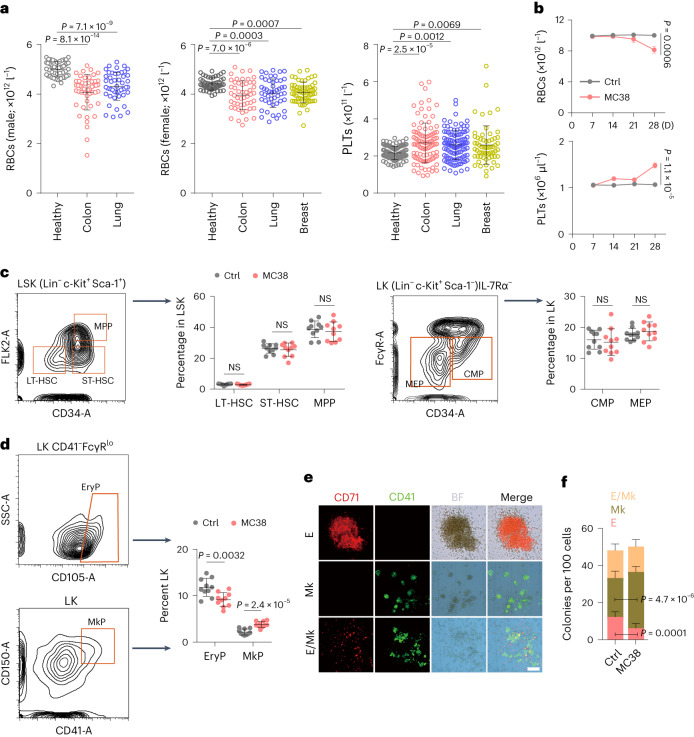
Advanced tumors induce abnormal MEP differentiation. **a**, Peripheral RBC and PLT analysis of healthy individuals (*n* = 50 males, *n* = 50 females) and individuals with untreated colon cancer (*n* = 50 males, *n* = 50 females), breast cancer (*n* = 60 females) and lung cancer (*n* = 50 males, *n* = 50 females). **b**–**d**, C57BL/6J mice were inoculated with MC38 colon cancer cells. RBCs and PLTs were counted once per week until MC38 tumors were approximately 2,000 mm^3^ at week 4 (**b**), then LT-HSCs, ST-HSCs, MPPs, CMPs and MEPs (**c**) and EryPs and MkPs (**d**) were analyzed after the last RBC and PLT count; Ctrl, control. **e**,**f**, The same as **b**, except that control and MC38 tumor-bearing mouse-derived MEPs were isolated and cultured in 10% collagen-based medium for 7 d. Colonies were labeled with anti-CD41 and anti-CD71 and were assessed by fluorescence microscopy (**e**). Representative images of mouse MEP-derived colonies (**e**) and results of a c.f.u. analysis of MEPs derived from control or MC38 tumor-bearing mice (**f**) are shown. Scale bar, 100 μm. BF, brightfield; E, erythrocyte; Mk, megakaryocyte. In **b**–**d** and **f**, *n* = 10 mice. Data were analyzed by two-tailed Student’s *t*-tests (**b**–**d** and **f**) or one-way analysis of variance (ANOVA) followed by a Bonferroni post hoc test (**a**). The data represent mean ± s.d. (**a**, **c**, **d** and **f**) or mean ± s.e.m. (**b**); NS, not significant. Source data

**Extended Data Fig. 1:**
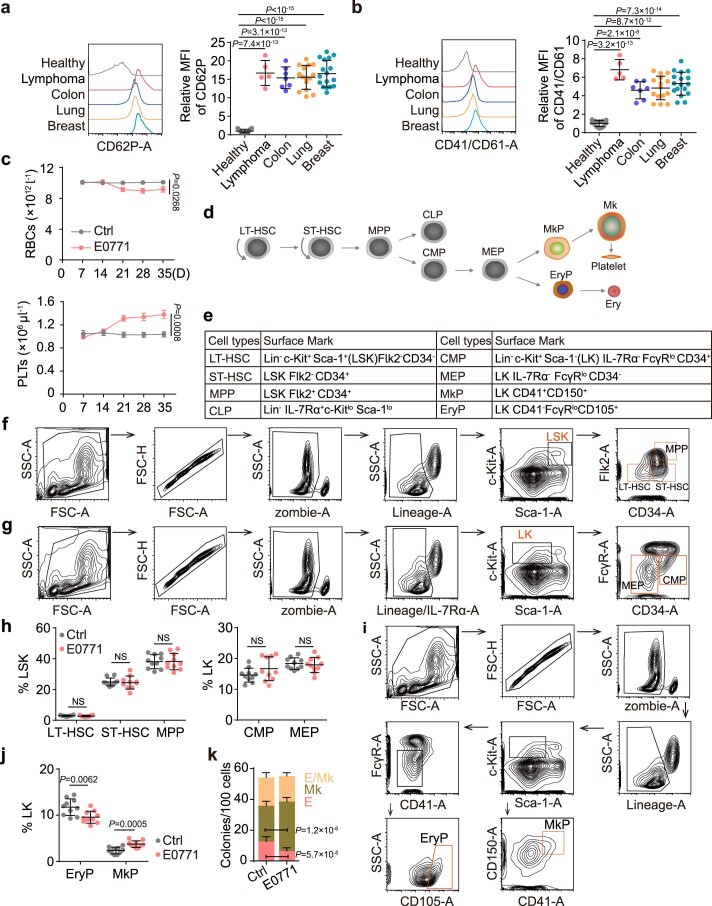
Abnormal MEP differentiation is induced by advanced tumors. **a**,**b**, The expression of P-selectin (CD62P) (**a**) and integrin GPIIb/IIIa (CD41/CD61) (**b**) in platelets of healthy donors (n = 12), lymphoma (n = 5), colon (n = 7), lung (n = 15), and breast (n = 17) cancer patients were determined by flow cytometry. **c**, C57BL/6 J mice were inoculated with E0771 breast cancer cells. Peripheral RBCs and PLTs of control or E0771 tumor bearing mice were counted every week until E0771 tumor approximately were 2000 mm^3^ at week 5. **d**, Classical model of the hematopoietic hierarchy. **e**, Hematopoietic stem cells and hematopoietic progenitor cells compartment can be distinguished by antigen markers, for instance, murine LT-HSC (Lin^−^Sca^−^1^+^C-kit^+^CD34^−^Flk2^−^), ST-HSC (Lin^−^Sca^−^1^+^C-kit^+^CD34^+^Flk2^−^), MPP (Lin^−^Sca^−^1^+^C-kit^+^CD34^+^Flk2^+^), CMP (Lin^−^IL-7Rα^-^Sca-1^-^C-kit^+^CD34^+^FcγR^lo^), MEP (Lin^−^IL-7Rα^-^Sca-1^-^C-kit^+^CD34^−^FcγR^lo^). **f**,**g**, Sorting strategies for murine LT-HSC, ST-HSC, MPP(**f**); CMP, MEP(**g**). **h**, C57BL/6 J mice were inoculated with E0771, flow cytometric analysis of LT-HSCs, ST-HSCs, MPPs, CMPs, MEPs at week 5. **i**, Sorting strategies for murine EryP, MkP. **j**,**k**, The experiment was the same as in **c**, except for flow cytometric analysis of EryPs, MkPs (**j**), then MEPs were isolated and cultured in 10% collagen-based medium for CFU analysis (**k**). In **c,h,j,k**, n = 10 mice. NS, not significant, Two^−^tailed Student’s t-test (**c**,**h**,**j**,**k**) or One-way ANOVA followed by Bonferroni’s test (**a**,**b**). The data represent mean ± s.d (**a**,**b**,**h**,**j**,**k**) or mean ± s.e.m. (**c**). Source data

Given the frequency of anemia and thrombocytosis in individuals with advanced cancer^1,2^, we analyzed the number of red blood cells (RBCs) and PLTs in individuals with stage III or IV colon (*n* = 100), lung (*n* = 99) or breast (*n* = 60) cancer. We found that numbers of RBCs and PLTs were reduced and elevated (Fig. 1a), respectively, in the blood compared to numbers observed in healthy control individuals. Analysis of P-selectin and integrin GPIIb/IIIa of PLTs revealed that these PLT-activating markers were upregulated in individuals with cancer (Extended Data Fig. 1a,b), suggesting that increased PLT activation contributes to tumor-associated events, such as metastasis and thrombosis. We then validated these clinical results in mice. After inoculating mouse MC38 colon cancer cells or E0771 breast cancer cells into C57BL/6 mice, we observed that PLTs and RBCs gradually increased and decreased, respectively, in the blood of mice bearing large tumors (approximately 2,000 mm^3^; Fig. 1b and Extended Data Fig. 1c). Hematopoietic stem cells (HSCs) differentiate from long-term HSCs (LT-HSCs) to short-term HSCs (ST-HSCs) and multipotent progenitors (MPPs); the MPPs then differentiate into common myeloid progenitors (CMPs) and downstream MEPs^25,26^ (Extended Data Fig. 1d). Flow cytometric analysis of bone marrow (BM) HSCs and progenitor cells (Extended Data Fig. 1e–g) revealed that the numbers of LT-HSCs, ST-HSCs, MPPs, CMPs and MEPs were not altered in tumor-bearing mice compared to in tumor-free control mice (Fig. 1c and Extended Data Fig. 1h). We thus postulated that MEPs biased their differentiation into megakaryocytes for thrombocytosis and erythrocytopenia. Megakaryocyte progenitor cells (MkPs) and erythrocyte progenitor cells (EryPs) are directly differentiated from MEPs to generate megakaryocytes and erythrocytes, respectively. Analysis of MkPs and EryPs by flow cytometry (Extended Data Fig. 1i) showed that MkPs increased and EryPs decreased in mice bearing colon or breast cancer (Fig. 1d and Extended Data Fig. 1j). By conducting a colony-forming unit (c.f.u.) assay^27^, we found that seeding normal MEPs in 10% collagen medium mainly formed CD41^+^CD71^+^ megakaryocyte/erythrocyte, CD71^+^ erythroid-only and CD41^+^ megakaryocyte-only colonies (Fig. 1e), whereas MEPs from tumor-bearing mice formed more megakaryocyte-only but less erythrocyte-only colonies (Fig. 1f and Extended Data Fig. 1k). Together, these results suggest that tumor growth gradually biases MEP differentiation into megakaryocytes, leading to thrombocythemia and anemia.

### AhR promotes MEP differentiation into megakaryocytes

**Fig. 2:**
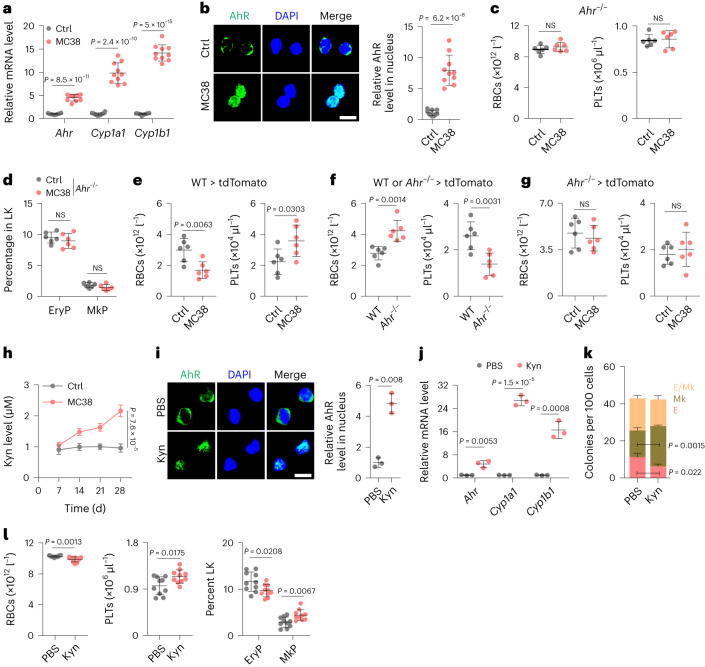
MEP differentiation is biased toward megakaryocytes by AhR activation. **a**,**b**, MEPs were isolated from control and MC38 tumor-bearing mice at week 4. Quantitative PCR with reverse transcription (RT–qPCR) detected *Ahr*, *Cyp1a1* and *Cyp1b1* expression (**a**). Cells were also stained for AhR and assessed by confocal microscopy (**b**). Scale bar, 10 μm. **c**,**d**, *Ahr*^–/–^ mice were inoculated with MC38 cells, and RBCs and PLTs were counted at a tumor size of approximately 2,000 mm^3^ (**c**). EryPs and MkPs were analyzed (**d**). **e**, tdTomato-transgenic mice were irradiated with 9 Gy by X-ray with 4-h intervals, and 10^6^ BM cells from WT mice were transferred into the irradiated mice. Mice were then inoculated with MC38 cells at week 6. Donor-derived CD41^+^tdTomato^−^ PLTs and ter119^+^tdTomato^−^ RBCs were counted at the same time point as in **c** and **d**. **f**,**g**, The same as **e**, except that WT or *Ahr*^–/–^ BM cells were transferred. Reconstituted peripheral blood RBCs and PLTs were analyzed at week 6 (**f**). Mice transferred with *Ahr*^–/–^ BM cells were inoculated with MC38 cells, and donor-derived PLTs and RBCs were analyzed at the same time point as in **c** and **d** (**g**). **h**, C57BL/6J mice were inoculated with MC38 cells, and serum Kyn levels from control and tumor-bearing mice were measured by high-performance liquid chromatography (HPLC) once per week for 4 weeks. **i**,**j**, MEPs were isolated from healthy C57BL/6J mice and treated with PBS or 5 μM Kyn. Treated MEPs were stained for AhR and analyzed by confocal microscopy at 48 h (**i**). Scale bar, 10 μm. Expression of *Ahr*, *Cyp1a1* and *Cyp1b1* was determined by RT–qPCR at 24 h (**j**). **k**, Analysis of c.f.u. for PBS- or Kyn-treated MEPs isolated from healthy C57BL/6J mice. **l**, C57BL/6J mice were treated with Kyn (400 μg i.p. every 12 h) for 2 weeks, followed by RBC, PLT, EryP and MkP analysis. In **a**, **b**, **h** and **l**, *n* = 10 mice. In **c**–**g**, *n* = 6 mice. In **i**–**k**, *n* = 3 independent experiments. Data were analyzed by two-tailed Student’s *t*-tests (**a**–**l**) and represent mean ± s.d (**a**–**g** and **i**–**l**) or mean ± s.e.m (**h**). Source data

**Extended Data Fig. 2:**
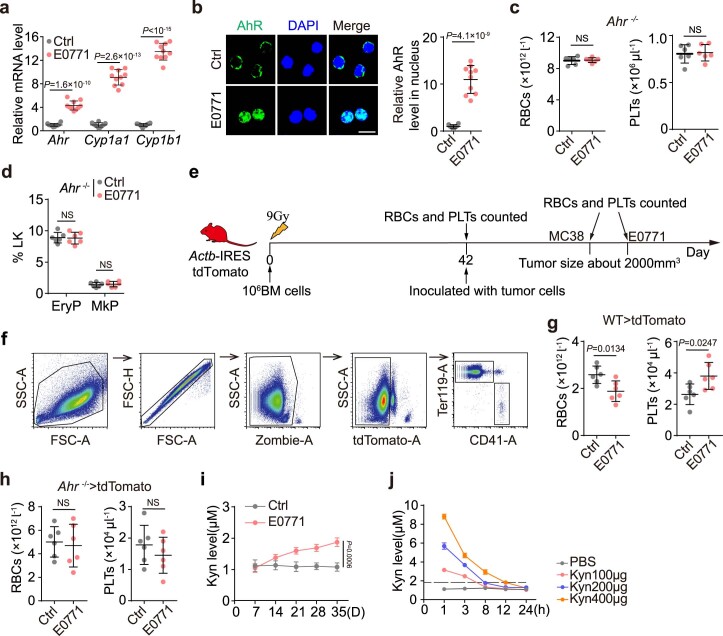
AhR regulates MEP differentiation into megakaryocytes. **a**,**b**, MEPs isolated from control and E0771 tumor bearing mice. The isolated cells were determined expression of *Ahr*, *Cyp1a1*, and *Cyp1b1* by RT-qPCR (**a**) and stained for AhR. Scale bar, 10 μm (**b**). **c**,**d**, *Ahr*^-/-^ mice were inoculated with E0771. RBCs and PLTs were counted at tumor size approximately 2000 mm^3^ (**c**), then analyzed EryPs and MkPs(**d**). **e**, Schematic representation of bone marrow transplantation model and treatment regimen. **f**-**h**, tdTomato transgenic mice irradiated and transferred with WT or *Ahr*^*-/-*^ murine BM cells, analysis strategies of donor-derived PLTs and RBCs as shown in (**f**); as show in **e**, irradiated tdTomato transgenic mice were inoculated with E0771 cells, WT mice-derived PLTs and RBCs were counted (**g**), *Ahr*^*-/-*^ mice-derived PLTs and RBCs were counted (**h**). **i**, C57BL/6 J mice were inoculated with E0771 cells. Serum Kyn levels of control and E0771 tumor bearing mice were measured by HPLC once a week for 5 weeks. **j**, Different doses of Kyn were injected to C57BL/6 J mice, then serum Kyn levels were detected at 1, 3, 8, 12, 24 hr by HPLC. In **a**,**b**,**i** n = 10 mice; In **c**,**d**,**g**,**h** n = 6 mice; In **j**, n = 5 mice. NS, not significant, Two^−^tailed Student’s t-test (**a**-**d**,**g**-**i**). The data represent mean ± s.d (**a**-**d**,**g**,**h**,**j**) or mean ± s.e.m (**i**). Source data

Next, we determined whether AhR plays a role in MEP differentiation. The genes encoding cytochrome P450 family 1 subfamily A polypeptide 1 (*CYP1A1*) and cytochrome P450 family 1 subfamily B polypeptide 1 (*CYP1B1*) are AhR-targeted genes^28,29^. The expression of both *Cyp1a1* and *Cyp1b1* was upregulated in MEPs isolated from MC38 or E0771 tumor-bearing mice compared to in control healthy mice (Fig. 2a and Extended Data Fig. 2a). In line with this finding, the active form of AhR in the nucleus of MEPs was verified by immunofluorescence staining (Fig. 2b and Extended Data Fig. 2b). However, tumor growth in *Ahr*^–/–^ mice did not affect MEP differentiation relative to that in wild-type (WT) mice, as evidenced by the consistent number of peripheral RBCs and PLTs (Fig. 2c and Extended Data Fig. 2c) and BM MkPs and EryPs (Fig. 2d and Extended Data Fig. 2d), suggesting that AhR regulates MEP differentiation into megakaryocytes in tumor-bearing mice. To verify this finding, we adoptively transferred BM cells to lethally irradiated (9 Gy) tdTomato-transgenic mice. Six weeks later, when the increased number of PLTs and RBCs stabilized, mice were inoculated with MC38 or E0771 tumor cells (Extended Data Fig. 2e). We found that TER119^+^tdTomato^−^ RBCs and CD41^+^tdTomato^−^ PLTs gradually decreased and increased, respectively, in tumor-bearing mice compared to in tumor-free control mice (Fig. 2e and Extended Data Fig. 2f,g). Intriguingly, the adoptive transfer of *Ahr*^–/–^ BM cells led to more RBCs and fewer PLTs in the periphery (Fig. 2f), which was not affected by tumor growth (Fig. 2g and Extended Data Fig. 2h). Kyn, a typical ligand for AhR, has been reported to be elevated in individuals with cancer and in tumor-bearing mice^20,24,30^. Consistently, higher serum Kyn levels were found in MC38 or E0771 tumor-bearing mice on day 14 after inoculation than in control mice (Fig. 2h and Extended Data Fig. 2i). Kyn treatment led to the entry of AhR into the nucleus and upregulation of *Cyp1a1* and *Cyp1b1* expression in MEPs (Fig. 2i,j). A c.f.u. assay showed that Kyn treatment biased MEP differentiation into megakaryocytes (Fig. 2k). To validate these results in vivo, we injected 400 μg of Kyn intraperitoneally (i.p.) into mice once every 12 h. This dosage imitated the Kyn content in tumor-bearing mice (Extended Data Fig. 2j). Two weeks later, we found that erythrocyte numbers were reduced, but the PLT count was elevated in the blood of treated mice, concomitant with increased MkPs but reduced EryPs (Fig. 2l). Together, these results suggest that increased AhR activity may unbalance the differentiation of MEPs into MkPs and EryPs, thus leading to thrombocytosis and erythropenia in tumor-bearing hosts.

### AhR upregulates RUNX1 to bias MEP differentiation

**Fig. 3:**
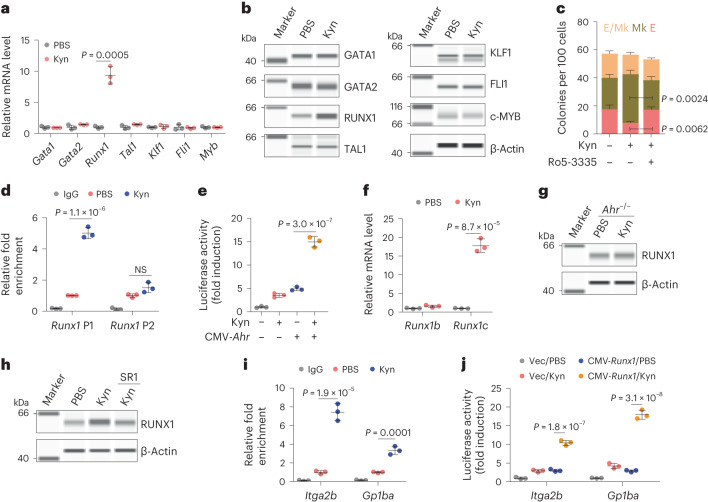
AhR regulates MEP differentiation bias by upregulating RUNX1. **a**, MEPs were isolated from healthy C57BL/6J mice and treated with Kyn. The expression of *Gata1*, *Gata2*, *Runx1*, *Tal1*, *Klf1*, *Fli1* and *Myb* was determined by RT–qPCR after 24 h. **b**, Protein expression was detected by digital western blotting after 48 h. **c**, The same as **a**, except that MEPs were treated with Kyn or Kyn combined with the RUNX1 inhibitor Ro5-3335 in collagen medium for 7 d, and a c.f.u. analysis was performed. **d**, Mouse MEPs were treated with PBS or Kyn for 48 h. CUT&RUN-qPCR analysis was performed with an antibody to AhR and *Runx1* P1 or P2 promotor-specific primers. **e**, NIH-3T3 cells were cotransfected with a *Runx1* P1 promoter-luciferase reporter PGL4.10 and pCMV6-*Ahr* plasmid for 24 h. Cells were then treated with Kyn for another 48 h, followed by analysis of luciferase activity. **f**, The same as **d**. *Runx1b* and *Runx1c* expression levels were detected by RT–qPCR. **g**, MEPs from *Ahr*^–/–^ mice were treated with Kyn for 48 h, and RUNX1 expression was determined by digital western blotting. **h**, Mouse MEPs were treated with Kyn or Kyn combined with SR1 for 48 h, and RUNX1 expression was determined by digital western blotting. **i**, Mouse MEPs were treated with PBS or Kyn for 72 h. CUT&RUN-qPCR analysis was performed with an antibody to RUNX1 and *Itga2b* or *Gp1ba* promotor-specific primers. **j**, NIH-3T3 cells were cotransfected with *Itga2b* or *Gp1ba* promoter-luciferase reporter PGL4.10 and pCMV6-*Runx1* plasmid for 24 h. Cells were then treated with Kyn for another 48 h, followed by analysis of luciferase activity; Vec, vehicle; CMV, cytomegalovirus. In **a**–**j**, *n* = 3 independent experiments. Data were analyzed by two-tailed Student’s *t*-tests (**a** and **f**) or one-way ANOVA followed by a Bonferroni post hoc test (**c**–**e**, **i** and **j**). Data represent mean ± s.d. Source data

**Extended Data Fig. 3:**
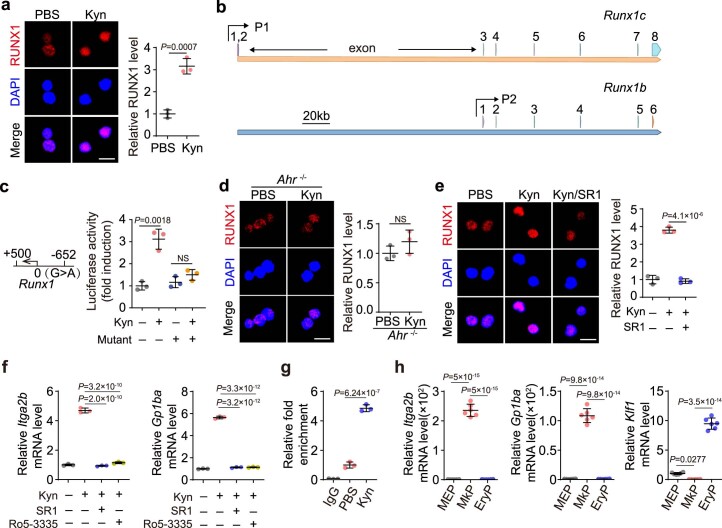
MEP differentiation bias is mediated by AhR-RUNX1 pathway. **a**, Murine MEPs were treated by Kyn for 48 hr, followed by RUNX1 staining. Scale bar, 10 μm. **b**, Schematic representations of the murine Runx1 P1 Promoter (top) and P2 promoter (bottom). **c**, NIH-3T3 was co-transfected with wild type or mutated *Runx1* promoter-luciferase reporter PGL4.10 and Ahr-overexpressing pCMV-6 for 24 hr. Cells were treated with Kyn for another 48 hr, followed by luciferase activity analysis. **d**, *Ahr*^*-/-*^ murine MEPs were treated by Kyn for 48 hr, followed by RUNX1 staining. Scale bar, 10 μm. **e**, Murine MEPs were treated by Kyn and SR1 for 48 hr, followed by RUNX1 staining. Scale bar, 10 μm. **f**, Murine MEPs were treated with Kyn (5 μM), Kyn combine SR1 (1 μM) or Kyn combined Ro5-3335 (2 μM) for 24 hr. RT-qPCR analysis was performed to detected the expression of *Itga2b* (left) or *Gp1ba* (right). **g**, Murine MEPs were treated with PBS or 5 μM Kyn for 48 hr. CUT&RUN-qPCR analysis was performed with an antibody to RUNX1 and *Klf1* promotor-specific primers. **h**, MEP, MkP and EryP were isolated from healthy C57BL/6 J mice. The expression of *Itga2b*, *Gp1ba* and *Klf1* in MEP, MkP and EryP were determined by RT-qPCR. In **a**,**c**-**g**, n = 3 independent experiments; In **h**, n = 6 mice. NS, not significant, Two-tailed Student’s t-test (**a**) or One-way ANOVA followed by Bonferroni’s test (**c-h**). The data represent mean ± s.d. Source data

Next, we investigated the molecular basis by which AhR-biased MEPs differentiate into megakaryocytes. A panel of transcription factors, such as GATA1, GATA2 and RUNX1, regulates MEP differentiation^11–14^. By examining these transcription factors in Kyn-treated MEPs, we found that expression of RUNX1, rather than the other transcription factors, was upregulated at the mRNA and protein levels (Fig. 3a,b), which was also verified by immunofluorescence (Extended Data Fig. 3a). Using a RUNX1 inhibitor, we found that Kyn-biased MEP differentiation was abrogated, but erythrocyte differentiation was augmented (Fig. 3c), prompting us to hypothesize that AhR regulates MEP differentiation via RUNX1. The *Runx1* gene possesses a distal P1 promoter and a proximal P2 promoter^31–33^ (Extended Data Fig. 3b), which results in two major isomers RUNX1c and RUNX1b with differences between their N-terminal amino acid sequences. Analysis of the JASPAR database revealed the AhR-binding core sequence (5′-GCGTG-3′) in the *Runx1* P1 and P2 promoters. By performing CUT&RUN-qPCR, we found that more DNA fragments were enriched in the P1 promoter than in the P2 promoter in Kyn-treated MEPs (Fig. 3d). A dual-luciferase assay also showed that AhR promoted reporter gene expression via the *Runx1* P1 promoter (Fig. 3e); however, this effect was abrogated by the mutation of 5′-GCGTG-3′ to 5′-GCATG-3′ in the P1 promoter (Extended Data Fig. 3c). In line with this finding, Kyn treatment upregulated the expression of *Runx1c* but not *Runx1b* (Fig. 3f). In addition, Kyn treatment did not alter RUNX1 expression in *Ahr*^–/–^ MEPs (Fig. 3g and Extended Data Fig. 3d). Consistently, pretreatment of MEPs with the AhR inhibitor SR1 abrogated the effect of Kyn on RUNX1 expression (Fig. 3h and Extended Data Fig. 3e). RUNX1 regulates a panel of target genes, such as *KLF1*, *ITGA2B* and *GP1BA*, to exert its function^34–36^. CUT&RUN-qPCR and dual-luciferase assays showed that Kyn treatment enables RUNX1 to bind and transactivate *Itga2b* and *Gp1ba* in MEPs (Fig. 3i,j). In addition, Kyn treatment upregulated the expression of *Itga2b* and *Gp1ba*, which, however, was abrogated by the AhR inhibitor SR1 and the RUNX1 inhibitor Ro5-3335 (Extended Data Fig. 3f), suggesting that AhR–RUNX1 regulates *Itga2b*/*Gp1ba* expression. Notably, *Klf1*, another target gene of RUNX1 (Extended Data Fig. 3g), although being not affected in Kyn-treated MEPs (Fig. 3a,b), was downregulated in MkPs, while *Itga2b* and *Gp1ba* were upregulated in MkPs, compared with in MEPs or EryPs (Extended Data Fig. 3h). Together, these data suggest that AhR transcriptionally activates RUNX1 to promote MEP differentiation into megakaryocytes.

### AhR upregulates SLC7A8 in MEPs to take up exogenous Kyn

**Fig. 4:**
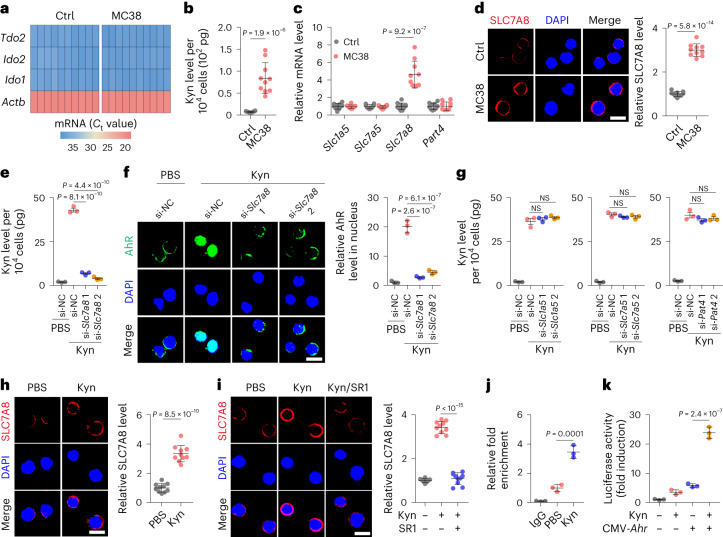
AhR promotes the uptake of Kyn in MEPs by upregulating SLC7A8. **a**–**d**, C57BL/6J mice were inoculated with MC38 cells, and MEPs were isolated from control and tumor-bearing mice at week 4. RT–qPCR cycling threshold (*C*_t_) values of *Actb*, *Ido1*, *Ido2* and *Tdo2* were determined (**a**), and intracellular Kyn levels of MEPs were determined by liquid chromatography–mass spectrometry (LC–MS; **b**). Expression of *Slc1a5*, *Slc7a5*, *Slc7a8* and *Pat4* in MEPs was detected by RT–qPCR (**c**), and MEPs were stained for SLC7A8 (**d**). Scale bar, 10 μm. **e**,**f**, MEPs transfected with negative control (NC) and *Slc7a8* siRNA (si1 or si2) were treated with PBS or Kyn for 48 h, intracellular Kyn levels were determined (**e**), and cells were stained for AhR (**f**). Scale bar, 10 μm. **g**, NC, *Slc1a5*, *slc7a5* and *Pat4* siRNA-transfected mouse MEPs were treated with PBS or Kyn for 48 h, and intracellular Kyn levels were determined. **h**, C57BL/6J mice were treated with PBS or Kyn for 2 weeks, and isolated MEPs were stained for SLC7A8. Scale bar, 10 μm. **i**, C57BL/6J mice were treated with Kyn or Kyn combined with SR1 (50 μg i.p. every day) for 2 weeks, and isolated MEPs were stained for SLC7A8. Scale bar, 10 μm. **j**, Mouse MEPs were treated with PBS or Kyn for 48 h, and CUT&RUN-qPCR was performed with antibody to AhR and promoter-specific primers against *Slc7a8*. **k**, NIH-3T3 cells expressing *Slc7a8* promoter-luciferase reporter PGL4.10 were cotransfected with pCMV6-*Ahr*. Cells were treated with Kyn for 48 h and analyzed by luciferase assay. In **a**–**d**, **h** and **i**, *n* = 10 mice. In **e**–**g**, **j** and **k**, *n* = 3 independent experiments. Data were analyzed by two-tailed Student’s *t*-tests (**b**–**d** and **h**) or one-way ANOVA followed by a Bonferroni post hoc test (**e**–**g** and **i**–**k**). Data represent mean ± s.d. Source data

**Extended Data Fig. 4:**
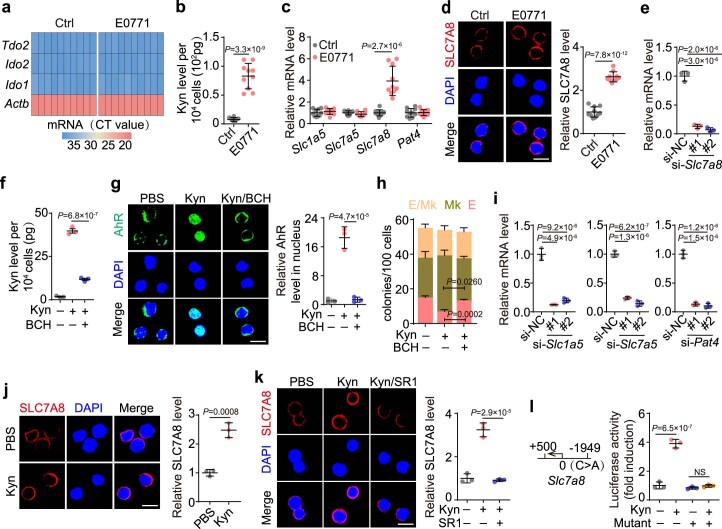
AhR upregulates SLC7A8 in MEPs to take up Kyn. **a**-**d**, C57BL/6 J mice were inoculated with E0771. MEPs were isolated from control and tumor bearing mice at week 5. RT-qPCR CT value of *Actb*, *Ido1*, *Ido2*, *Tdo2* were determined (**a**); intracellular Kyn level per 10^4^ MEPs were detected by LC-MS (**b**), *Slc1a5*, *Slc7a5*, *Slc7a8* and *Pat4* expression were determined by RT-qPCR (**c**), MEPs were stained for SLC7A8. Scale bar, 10 μm (**d**). **e**, Murine MEPs were treated with *Slc7a8*-targeted siRNAs, interference efficiency of siRNAs was determined by RT-qPCR at 24 hr, **f**, Murine MEPs were treated with Kyn or Kyn combine with BCH for 48 hr, intracellular Kyn per 10^4^ MEPs was measured. **g**, The same as **f**, except that MEPs were stained for AhR. scale bar, 10 μm. **h**, The same as **f**, except that MEPs were cultured in collagen-based medium for 7 days, followed by CFU analysis. **i**, Murine MEPs were treated with *Slc1a5*, *Slc7a5* or *Pat4*-targeted siRNA for 48 hr, *Slc1a5, Slc7a5*, *Pat4* expression levels were detected by RT-qPCR. **j**, Murine MEPs were treated with PBS or Kyn for 48 hr. Cell was stained for SLC7A8. Scale bar, 10 μm. **k**, Murine MEPs were treated with Kyn or Kyn combine SR1 for 48 hr. Cells was stained for SLC7A8. Scale bar, 10 μm. **l**, NIH-3T3 was co-transfected with wild type or mutated *Slc7a8* promoter-luciferase reporter PGL4.10 and *Ahr*-overexpressing pCMV-6 for 24 hr. Cells were treated with Kyn for another 48 hr, followed by luciferase activity analysis. In **a-d** n = 10 mice; In **e**-**l**, n = 3 independent experiments. NS, not significant, Two-tailed Student’s t-test (**b**-**d**,**j**) or One-way ANOVA followed by Bonferroni’s test (**e**-**i**,**k**,**l**). The data represent mean ± s.d. Source data

Next, we investigated the manner by which AhR was activated in MEPs. Kyn is a typical endogenous ligand, which is produced by the catalysis of tryptophan via indoleamine 2,3-dioxygenase 1 (IDO1), IDO2 or tryptophan 2,3-dioxygenase 2 (TDO2)^20,30^. Surprisingly, although *Ido1*, *Ido2* and *Tdo2* were barely expressed in MEPs (Fig. 4a and Extended Data Fig. 4a), a high level of Kyn was found in the MEPs of tumor-bearing mice and a very low Kyn level was present in the MEPs of tumor-free mice (Fig. 4b and Extended Data Fig. 4b), suggesting that MEPs from tumor-bearing hosts might use exogenous Kyn to activate AhR. In fact, tumor tissues release large amounts of Kyn to the periphery, which can be used by MEPs to activate AhR. To achieve this, MEPs have to express transporters that transport Kyn across the plasma membrane. SLC1A5, SLC7A5, SLC7A8 and PAT4 have shown the ability to mediate Kyn transportation^23,37^. We found that although MEPs expressed the four transporters, only SLC7A8 was upregulated in the MEPs of tumor-bearing mice (Fig. 4c,d and Extended Data Fig. 4c,d). The use of *Slc7a8* short interfering RNAs (siRNAs) or the inhibitor 2-amino-2-norbornanecarboxylic acid (BCH) blocked the entry of Kyn into MEPs (Fig. 4e and Extended Data Fig. 4e,f), concomitant with a reduction in AhR nuclear localization (Fig. 4f and Extended Data Fig. 4g). As expected, BCH treatment reversed the biased differentiation of MEPs (Extended Data Fig. 4h). However, knockdown of *Slc1a5*, *Slc7a5* or *Pat4* had minor effects on the transport of Kyn into MEPs (Fig. 4g and Extended Data Fig. 4i). Notably, in the in vitro assay, we found that the addition of Kyn markedly upregulated SLC7A8 expression in MEPs (Extended Data Fig. 4j), which was also verified in Kyn-treated mice (Fig. 4h), prompting us to postulate a positive feedback mechanism through which SLC7A8 was upregulated by Kyn-activated AhR. Indeed, SR1 effectively inhibited Kyn-induced SLC7A8 upregulation in MEPs both in vitro and in vivo (Fig. 4i and Extended Data Fig. 4k). A CUT&RUN assay also showed that AhR bound to the *Slc7a8* promoter (Fig. 4j), and a luciferase assay verified that AhR promoted *Slc7a8* expression (Fig. 4k and Extended Data Fig. 4l). Together, these data suggest that AhR transactivates the Kyn transporter gene and promotes the uptake of Kyn by MEP.

### Kyn–AhR–RUNX1 biases MEP differentiation in vivo

**Fig. 5:**
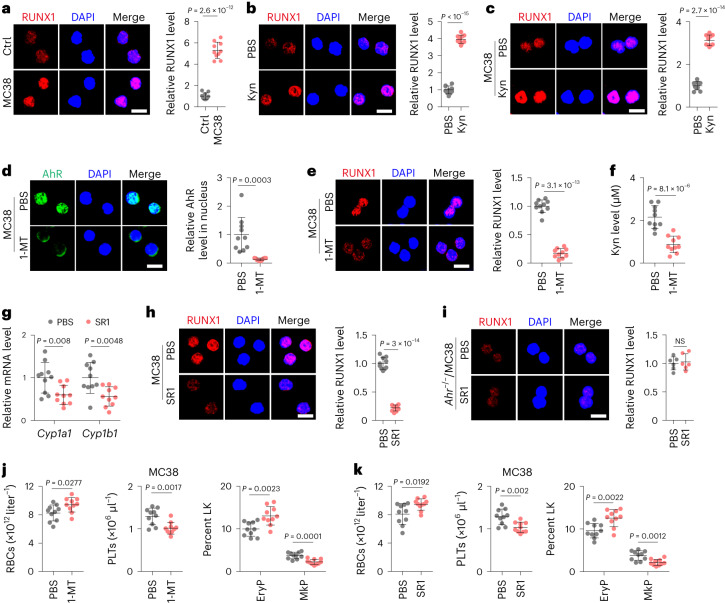
The Kyn–AhR–RUNX1 pathway regulates MEP differentiation into megakaryocytes in vivo. **a**, C57BL/6J mice were inoculated with MC38 cells, and MEPs were isolated and stained for RUNX1 at week 4. Scale bar, 10 μm. **b**, C57BL/6J mice were treated with PBS or Kyn for 2 weeks, and MEPs were isolated and stained for RUNX1; scale bar, 10 μm. **c**, The same as **a**, except that tumor-bearing mice were treated with PBS or Kyn for 2 weeks. Isolated MEPs were stained for RUNX1. Scale bar, 10 μm. **d**–**f**, The same as **a**, except that tumor-bearing mice were treated PBS or 1-MT (5 mg ml^−1^ in drinking water, 3–4 ml per mouse per day) for 2 weeks. MEPs were isolated and stained for AhR (**d**) and RUNX1 (**e**). Scale bar, 10 μm. Serum Kyn levels of PBS- or 1-MT-treated mice were measured by HPLC (**f**). **g**,**h**, The same as **a**, except that MC38 tumor-bearing mice were treated with PBS or SR1 for 2 weeks. *Cyp1a1* and *Cyp1b1* expression in MEPs was determined by RT–qPCR (**g**), and MEPs were stained for RUNX1 (**h**). **i**, *Ahr*^–/–^ mice were inoculated with MC38 cells, tumor-bearing mice were treated with PBS or SR1, and MEPs were isolated and stained for RUNX1. Scale bar, 10 μm. **j**, The same as **d**, except that RBCs and PLTs or EryPs and MkPs in BM were analyzed. **k**, The same as **g**, except that RBCs and PLTs or EryPs and MkPs were analyzed. In **a**–**h**, **j** and **k**, *n* = 10 mice. In **i**, *n* = 6 mice. Data were analyzed by two-tailed Student’s *t*-tests (**a**–**k**) and represent mean ± s.d. Source data

**Extended Data Fig. 5:**
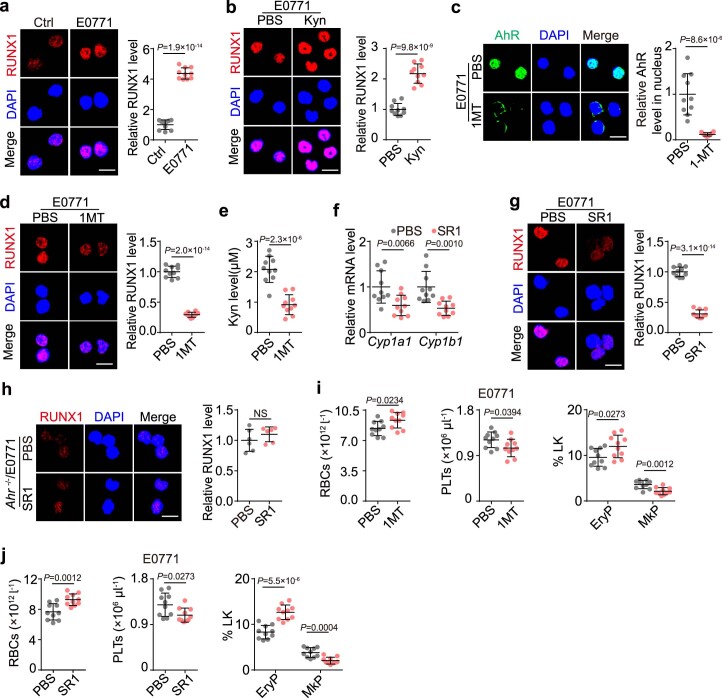
In vivo MEP differentiation is regulated by Kyn-AhR-RUNX1 axis. **a**, C57BL/6 J mice were inoculated with E0771. MEPs were isolated and stained for RUNX1 at week 5. Scale bar, 10 μm. **b**, The same as **a**, except that E0771 tumor bearing mice were injected with PBS or Kyn for 2 weeks, MEPs were isolated and stained for RUNX1. Scale bar, 10 μm. **c**-**e**, The same as **a**, except that E0771 tumor bearing mice were treated with PBS and 1MT for 2 weeks, MEPs were isolated and stained for AhR (**c**), RUNX1 (**d**), Scale bar, 10 μm. Serum Kyn level of treated mice was measured by HPLC (**e**). **f**, **g**, The same as **a**, except that E0771 tumor bearing mice were injected with PBS or SR1 for 2 weeks. *Cyp1a1*, *Cyp1b1* expression in MEPs were detected by RT-qPCR (**f**) and MEPs were stained for RUNX1. Scale bar, 10 μm(**g**). **h**, AhR ^-/-^mice were inoculated with E0771. Then tumor bearing mice were administrated with PBS or SR1, MEPs were isolated and stained for RUNX1. scale bar, 10 μm. **i**, The same as **c**, except that peripheral RBCs and PLTs or EryPs and MkPs in BM were analyzed. **j**, The same as **f**, except that peripheral RBCs and PLTs or EryPs and MkPs were counted. In **a-g**,**i**,**j**, n = 10 mice; In **h**, n = 6 mice; NS, not significant, Two-tailed Student’s t-test (**a**-**j**). The data represent mean ± s.d. Source data

Next, we investigated whether our experimental results could be confirmed in vivo. In addition to AhR, which was highly active in the MEPs of tumor-bearing mice, we determined RUNX1 levels. We found that tumor-bearing mice displayed higher levels of RUNX1 in MEPs (Fig. 5a and Extended Data Fig. 5a). Injection of Kyn (i.p.) augmented RUNX1 expression in both healthy and tumor-bearing mice (Fig. 5b,c and Extended Data Fig. 5b). Given Kyn production via IDO1, we used 1-MT, an IDO1 inhibitor, to treat tumor-bearing mice. As expected, 1-MT effectively blocked AhR nuclear translocation (Fig. 5d and Extended Data Fig. 5c) and downregulated RUNX1 expression in MEPs (Fig. 5e and Extended Data Fig. 5d), concomitant with decreased Kyn levels in peripheral blood (Fig. 5f and Extended Data Fig. 5e). In addition, AhR inhibition by SR1 downregulated *Cyp1a1*, *Cyp1b1* and *Runx1* expression in MEPs in tumor-bearing mice (Fig. 5g,h and Extended Data Fig. 5f,g). However, RUNX1 downregulation was not detected in *Ahr*^–/–^ tumor-bearing mice (Fig. 5i and Extended Data Fig. 5h). Given the role of RUNX1 in the regulation of megakaryocyte differentiation, we analyzed MEP differentiation under conditions of IDO1 or AhR inhibition. The results showed that both 1-MT and SR1 hindered MkP generation but favored EryP production in tumor-bearing mice (Fig. 5j,k and Extended Data Fig. 5i,j). Together, these results suggest that Kyn, via the AhR–RUNX1 pathway, leads to abnormal MEP differentiation, thrombocytosis and anemia in vivo.

### Kyn–AhR–RUNX1 mediates thrombocytosis in humanized mice

**Fig. 6:**
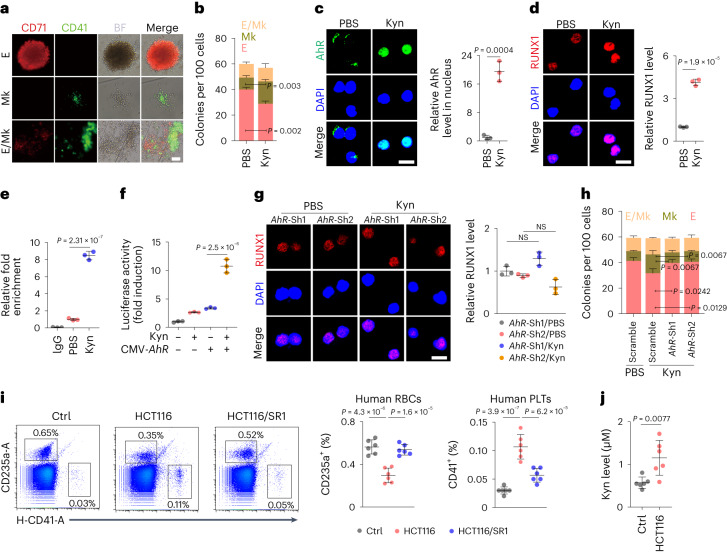
Thrombocytosis and erythropenia are induced by Kyn in humanized mice. **a**, Representative images of human MEP-derived colonies. Scale bar, 100 μm. **b**, MEPs from human UCB were cultured in collagen-based medium and treated with PBS or Kyn for 12 d, followed by c.f.u. analysis. **c**,**d**, Human MEPs were treated with PBS or Kyn for 48 h, and cells were stained for AhR (**c**) or RUNX1 (**d**). Scale bar, 10 μm. **e**, CUT&RUN-qPCR analysis was performed with anti-AhR and *RUNX1* P1 promoter-specific primers. **f**, HEK 293T cells were cotransfected with *RUNX1* P1 promoter-luciferase reporter PGL4.10 and AhR-overexpressing plasmid for 24 h. Cells were then treated with Kyn for another 48 h, followed by analysis of luciferase activity. **g**, Human MEPs transfected with short hairpin RNA (shRNA) targeting *Ahr* (Sh1 and Sh2) were treated with Kyn for 48 h and stained for RUNX1. Scale bar, 10 μm. **h**, Human MEPs were transfected with scrambled shRNA and shRNA targeting *Ahr* (Sh1 and Sh2), cultured in collagen medium and treated with Kyn for c.f.u. analysis at day 12. **i**, HCT116 tumor-bearing humanized mice were treated with PBS and SR1 for 2 weeks, and human-derived RBCs and PLTs in peripheral blood were analyzed. **j**, The same as **i**, except that serum Kyn levels of healthy control and tumor-bearing humanized mice were measured by HPLC. In **i** and **j**, *n* = 6 mice. In **b**–**h**, *n* = 3 independent experiments. Data were analyzed by two-tailed Student’s *t*-tests (**b**–**d** and **j**) or one-way ANOVA followed by a Bonferroni post hoc test (**e**–**i**). Data represent mean ± s.d. Source data

**Extended Data Fig. 6:**
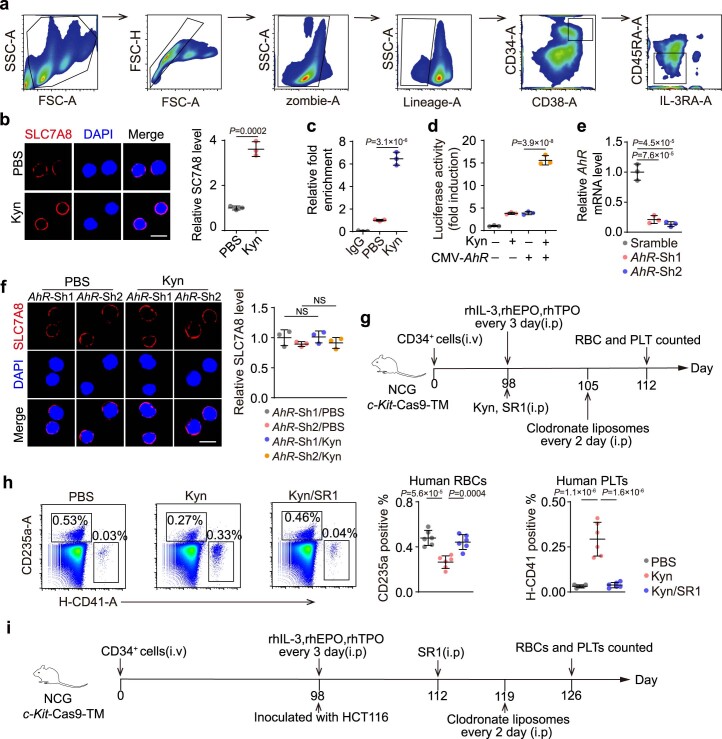
Kyn promotes thrombocytosis and erythropenia in humanized mice. **a**, Human MEP sorting strategy. **b**, Human MEPs were treated with Kyn for 48 hr. Cells were stained for SLC7A8. Scale bar, 10 μm. **c**, The same as **b**, except that CUT&RUN-qPCR analysis was performed with AhR antibody and *SLC7A8* promoter-specific primers. **d**, 293 T was co-transfected with *SLC7A8* promoter-luciferase reporter PGL4.10 and *AhR*-overexpressing pCMV-6 for 24 hr. Cells were treated with Kyn for another 48 hr, followed by luciferase activity analysis. **e**,**f**, Human MEPs were transfected with *AhR*-targeted ShRNA (Sh1 or Sh2), AhR expression level was detected by RT-qPCR at 24 hr (**e**); *AhR* ShRNA (Sh1 or Sh2)-human MEPs were treated with PBS or Kyn for 48 h. Cells were stained for SLC7A8. scale bar, 10 μm (**f**). **g**, Humanized mouse model establishment and treatment Diagram. **h**, Humanized mice were treated with Kyn or SR1 combined with Kyn as shown in **g**, followed by flow cytometry analysis of peripheral human derived-RBCs and PLTs. **i**, Schematic representation of humanized mouse model and treatment regimen. In **h**, n = 6 mice; In **b**-**f**, n = 3 independent experiments. NS, not significant, Two-tailed Student’s t-test (**b**) or One-way ANOVA followed by Bonferroni’s test (**c**-**f**,**h**). The data represent mean ± s.d. Source data

Next, we investigated whether Kyn-biased mouse MEP differentiation could be translated into human MEPs. To this end, we isolated CD34^+^CD38^+^IL-3RA^−^CD45RA^−^ MEPs from human umbilical cord blood (UCB; Extended Data Fig. 6a) and treated the cells with 10% collagen medium, which resulted in c.f.u. formation (Fig. 6a), consistent with previous reports^38,39^. After Kyn treatment, we found that Kyn facilitated differentiation toward MkPs in the c.f.u. assay (Fig. 6b), consistent with the differentiation of mouse MEPs by Kyn. Moreover, immunofluorescence staining showed that Kyn treatment resulted in the translocation of AhR into the nucleus of human MEPs (Fig. 6c) and upregulation of RUNX1 and SLC7A8 expression (Fig. 6d and Extended Data Fig. 6b). To further verify that the Kyn–AhR pathway is used by human MEPs to upregulate RUNX1 and SLC7A8 expression, we performed CUT&RUN analyses of Kyn-treated human MEPs. As expected, AhR-enriched DNA fragments from the *RUNX1* P1 promoter were detected and enhanced by Kyn treatment (Fig. 6e). Luciferase assays showed that AhR bound to the *RUNX1* P1 promoter and promoted reporter gene expression (Fig. 6f). Similar experiments showed that SLC7A8 expression was regulated by AhR (Extended Data Fig. 6c,d). Consistent with this, Kyn had little effect on the expression of RUNX1 in *AhR*-knockdown human MEPs (Fig. 6g and Extended Data Fig. 6e). In addition, Kyn did not affect SLC7A8 expression in these MEPs (Extended Data Fig. 6f), consistent with the positive feedback between AhR and SLC7A8 (refs. ^23,40^) by which SLC7A8 transports Kyn to activate AhR and the latter in turn promotes SLC7A8 expression. By conducting a c.f.u. assay, we found that although Kyn facilitated differentiation toward MkPs, *Ahr* knockdown switched differentiation toward EryPs (Fig. 6h). To validate the in vitro human results in vivo, we constructed a humanized mouse model by transplanting CD34^+^ UBCs into severely immunodeficient NCG-X mice, which lack the *Kit* gene and support human HSC transplantation without radiation^41,42^. These humanized mice were treated with Kyn or Kyn plus SR1 in combination with chlodronate (Extended Data Fig. 6g), which can delete macrophages to avoid phagocytosis of the generated human PLTs and RBCs^43,44^. As expected, Kyn treatment increased and decreased the amount of human PLTs and RBCs, respectively, which was blunted by SR1 (Extended Data Fig. 6h). By inoculating HCT116 human colon cancer cells into mice with treatment (Extended Data Fig. 6i), we found that the amounts of PLTs and RBCs also increased and decreased, respectively, in conjunction with tumor growth and elevated serum Kyn levels, which were also blunted by SR1 treatment (Fig. 6i,j). Together, these results suggest that the AhR–RUNX1 pathway triggered by Kyn decreases erythrocyte counts but increases PLT counts in cancer-bearing hosts.

### Kyn induces abnormal MEP differentiation in individuals with cancer

**Fig. 7:**
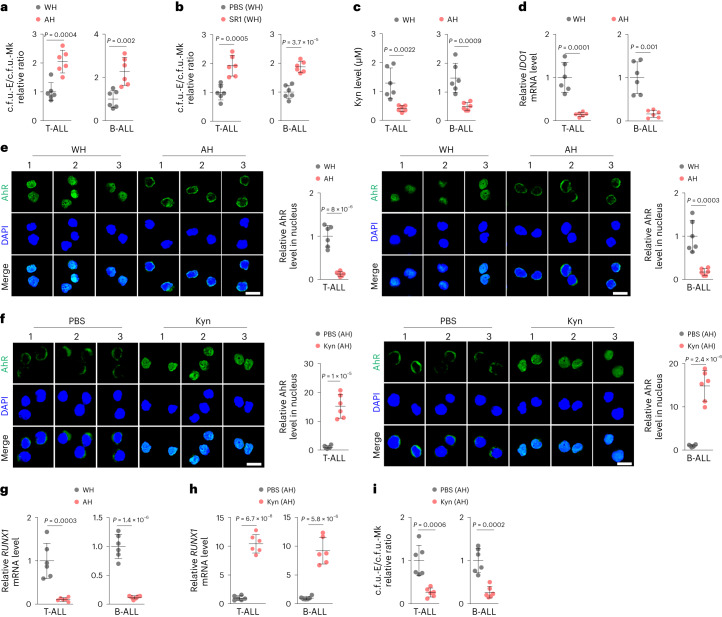
AhR mediates abnormal MEP differentiation in individuals with cancer. **a**, MEPs were isolated from the BM of individuals with T-ALL (*n* = 12; without HSCT (WH) *n* = 6, after HSCT (AH) *n* = 6) and B-ALL (*n* = 12; without HSCT *n* = 6, with HSCT *n* = 6), followed by c.f.u. analysis; c.f.u.-E, erythroid only; c.f.u.-Mk, megakaryocyte only. **b**, MEPs from individuals without HSCT were treated with PBS or SR1, followed by c.f.u. analysis. **c**, BM Kyn levels in individuals with cancer were detected by HPLC. **d**, BM-derived mononuclear cells from individuals with cancer were isolated, and *IDO1* expression was detected by RT–qPCR. **e**, MEPs were isolated from the BM of individuals with T-ALL and B-ALL and were stained for AhR. Scale bar, 10 μm. **f**, MEPs from individuals who had received HSCT were treated with Kyn for 48 h, and cells were stained for AhR. Scale bar, 10 μm. **g**, MEPs were isolated from the BM of individuals with T-ALL and B-ALL, and *RUNX1* expression was detected by RT–qPCR. **h**, MEPs isolated from individuals who had received HSCT were treated with Kyn for 24 h, and *RUNX1* expression was detected by RT–qPCR. **i**, MEPs isolated from individuals after HSCT (T-ALL, *n* = 6; B-ALL, *n* = 6) were treated with PBS or Kyn in collagen medium, followed by c.f.u. analysis. Data were analyzed by two-tailed Student’s *t*-tests (**a**–**i**) and represent mean ± s.d. Source data

**Extended Data Fig. 7:**
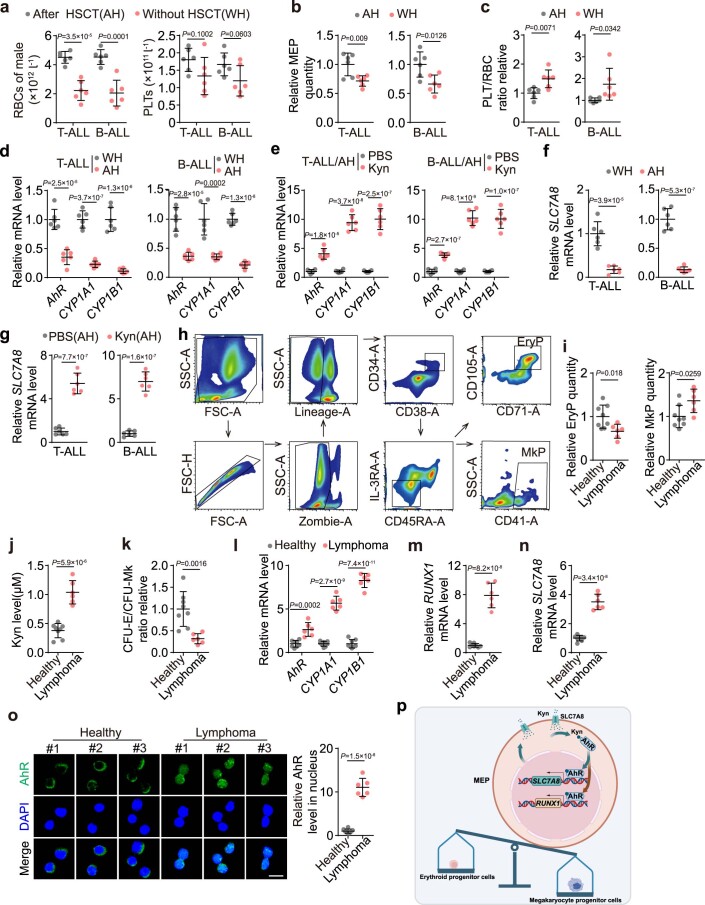
Abnormal MEP differentiation in cancer patients is mediated by AhR. **a-g**, Peripheral RBCs (left) and PLTs (right) analysis of newly diagnosis (n = 6) and HSCT (n = 6) T-ALL patients, or newly diagnosis (n = 6) and HSCT (n = 6) B-ALL patients (**a**); MEPs quantity in bone marrow aspiration sample of T-ALL (left) or B-ALL (right) patients with or without HSCT were analyzed (**b**); Platelet to red blood cell ratio of T-ALL (left) or B-ALL (right) patients with or without HSCT were analyzed (**c**); MEPs were isolated from T-ALL and B-ALL patients. *AhR*, *CYP1A1*, *CYP1B1* expression were detected by RT-qPCR (**d**); MEPs from patients after HSCT were treated with Kyn for 24 hr. *AhR*, *CYP1A1*, *CYP1B1* expression levels were detected by RT-qPCR (**e**); The same as **d**, except that *SLC7A8* was detected by RT-qPCR (**f**); The same as **e**, except that *SLC7A8* was detected by RT-qPCR (**g**). **h**, Human EryP and MkP sorting strategy. **i**, Human megakaryocytes precursors and erythroprogenitors from healthy donors (n = 8) and lymphoma patients (n = 6) without tumor cell infiltration into the bone marrow were analyzed by flow cytometry. **j**, HPLC detected bone marrow Kyn levels of healthy donors (n = 8) and lymphoma patients (n = 6). **k**, MEPs from BM of donors (n = 8) and lymphoma patients (n = 6) were performed for CFU analysis. **l**-**n**, *AhR*, *CYP1A1*, *CYP1B1* (**l**), *RUNX1* (**m**), and *SLC7A8* (**n**) expression of donors (n = 8) and lymphoma patients (n = 6) derived-MEPs were detected by RT-qPCR. **o**, MEPs from donors and lymphoma patients were stained for AhR. scale bar, 10 μm. **p**, The schematic diagram of MEP differentiation into MkP. Two-tailed Student’s t-test (**a**-**g**,**i-o**). The data represent mean ± s.d. Source data

Finally, we attempted to validate our findings by using clinical samples. BM cells were collected from individuals with T cell acute lymphoblastic leukemia (T-ALL; *n* = 12) and B cell acute lymphoblastic leukemia (B-ALL; *n* = 12) who had not received treatment before or who had received HSC transplantation (HSCT; *n* = 6 for each type). Participant information is presented in Supplementary Table 5. Numbers of both RBCs and PLTs of newly diagnosed individuals were lower than those of individuals who had received HSCT (Extended Data Fig. 7a). Notably, although the numbers of MEPs decreased in newly diagnosed individuals compared to that observed in individuals who had received HSCT (Extended Data Fig. 7b), the ratio of PLTs to RBCs was higher before HSCT (Extended Data Fig. 7c). Using isolated MEPs to perform a c.f.u. assay, we found that MEPs from untreated individuals biased the differentiation to megakaryocytes more than those from HSCT-treated individuals (Fig. 7a); however, this was disrupted by the addition of SR1 (Fig. 7b). In line with this finding, Kyn levels were much lower in the BM of individuals who had received HSCT (Fig. 7c), concomitant with the downregulation of *IDO1* in BM cells (Fig. 7d). To validate that the effect of Kyn is mediated by AhR, we determined AhR activity in MEPs from individuals with T-ALL or B-ALL. As expected, we found that HSCT resulted in the downregulation of *AhR*, *CYP1A1* and *CYP1B1* expression in MEPs, concomitant with decreased nuclear localization of AhR (Fig. 7e and Extended Data Fig. 7d), whereas the addition of Kyn blocked the HSCT-induced effects (Fig. 7f and Extended Data Fig. 7e). In line with this finding, *RUNX1* expression was downregulated in MEPs of individuals who had received HSCT (Fig. 7g). In addition, expression of the gene encoding the Kyn transporter SLC7A8 was downregulated in MEPs (Extended Data Fig. 7f). However, Kyn treatment upregulated *RUNX1* and *SLC7A8* expression in MEPs from individuals who had received HSCT (Fig. 7h and Extended Data Fig. 7g) and biased MEP differentiation into megakaryocytes (Fig. 7i). In addition, BM samples were collected from individuals with lymphoma (*n* = 6) without tumor cell infiltration. We found that more MkPs and fewer EryPs were present in the BM of individuals with lymphoma than in the BM of healthy donors (*n* = 8; Extended Data Fig. 7h,i). Consistently, BM Kyn levels were higher (Extended Data Fig. 7j), and the differentiation of MEPs was more biased toward megakaryocytes than observed in healthy donors (Extended Data Fig. 7k). In addition, the expression of *AhR*, *CYP1A1*, *CYP1B1*, *RUNX1* and *SLC7A8* was upregulated in MEPs of individuals with lymphoma (Extended Data Fig. 7l–n), concomitant with enhanced nuclear localization of AhR (Extended Data Fig. 7o). Together, these results suggest that the Kyn–AhR–RUNX1 pathway also mediates abnormal differentiation of MEPs in individuals with cancer (Extended Data Fig. 7p).

## Discussion

Thrombocytosis and anemia are common in individuals with cancer^1,45^. Here we identified a tumor release factor-mediated pathway that causes thrombocytosis and anemia. During tumor growth, large amounts of Kyn are released to the periphery, enter MEPs and bias their differentiation into megakaryocytes by activating AhR. Tryptophan is an essential amino acid widely used by tumor cells, at least partially due to the Myc oncoprotein, which can induce the expression of the tryptophan transporters SLC7A5 and SLC1A5 (ref. ^46^). Thus, tumor cells take up and catalyze tryptophan for Kyn production via IDOs. The released Kyn then activates AhR in MEPs. In line with our study, high levels of the AhR ligand TCDD increase the number of PLTs in the blood of exposed individuals^47^ and Kyn-induced anemia during inflammation^48^.

MEPs are bipotential with a fate decision to the megakaryocyte versus erythrocyte lineage, which, however, is not completely understood. In this study, we identified that exogenous Kyn is critical for regulating MEP fate decisions in individuals with cancer. However, MEPs do not seem to generate endogenous Kyn, in which the tryptophan-degrading enzymes are rarely detected, raising the question of how MEPs effectively take up Kyn. We demonstrated that SLC7A8 is the main transporter that transports Kyn into MEPs. Notably, this uptake pathway is regulated by a positive feedback loop, because Kyn-activated AhR upregulates SLC7A8 expression in MEPs. Thus, activated AhR is present in MEPs in individuals with cancer, which provides new insights into MEP fate decisions. After activation by GATA1, the transcription factor KLF1/EKLF promotes erythroid differentiation and suppresses megakaryocytic differentiation. By contrast, FLI1 directly activates genes, such as *ITGA2B*, *GP1BA* and *GPIX*, for megakaryocyte maturation. Notwithstanding this transcriptional regulation, Kyn–AhR was shown to transactivate RUNX1, another key transcription factor for megakaryocytic lineage differentiation. By performing single-cell c.f.u. assays, we clearly demonstrate that exogenous Kyn biases MEP differentiation to the megakaryocyte lineage through the AhR–RUNX1 pathway, which is likely to play an instructive role. This is unlike thrombopoietin (TPO) and erythropoietin (EPO), which play permissive roles in megakaryopoiesis and erythropoiesis, respectively. In addition, AhR seems to have a permissive effect, considering its regulating the polyploidization of megakaryocytes^18^. Whether Kyn prejudicially targets MEPs rather than other hematopoietic progenitors due to the differential expression of SLC7A8 is worthy of investigation in the future.

The present study has several important clinical implications. Kyn concentration may range from 0.1 to 0.5 μM in normal blood but increases to up to 1 μM in individuals with cancer, which has pathophysiological effects by activating AhR of target cells^23^. To recapitulate this, we used a humanized mouse model to conduct MEP differentiation experiments with or without Kyn treatment. Moreover, we verified the Kyn–AhR–RUNX1 pathway in MEPs freshly isolated from individuals with leukemia. It is feasible to obtain BM from individuals with leukemia; however, we cannot obtain BM from individuals with solid tumors because of ethical issues. Nevertheless, leukemic and solid tumor cells highly mobilize the Kyn–AhR pathway, leading to the fidelity of MEP differentiation among individuals with various tumors. Apart from individuals with cancer, our findings might be useful in explaining thrombocytosis in individuals without cancer with the JAK2-V617F mutation^49^. This mutation leads to the activation of JAK2. However, activated JAK2 may result in the upregulation of IDO1 expression via activated STAT1/STAT2, thus producing more endogenous Kyn to bias MEP differentiation into megakaryocytes. Whether this JAK2 mutation drives thrombocytosis through the IDO–Kyn pathway is currently under study. Overall, this study identified that AhR activation drives abnormal erythrocyte and PLT differentiation in individuals with cancer, and targeting the Kyn–AhR pathway could be explored to treat anemia and thrombocytosis in individuals with cancer.

## Methods

### Cell lines

Mouse tumor cell lines MC38 (colon cancer) and E0771 (breast cancer), the mouse embryonic fibroblast cell line NIH-3T3, the human tumor cell line HCT116 (colon cancer) and the human embryonic kidney cell line HEK 293T were purchased from the China Center for Type Culture Collection and were cultured in RPMI 1640 medium (Gibco) or DMEM medium (Gibco) with 10% fetal bovine serum (Gibco). Before the study, the cells were examined for the presence of *Mycoplasma*, checked for interspecies contamination and validated using isoenzyme and short tandem repeat studies in the Cell Resource Centre of Peking Union Medical College.

### Mouse transplant tumor models

C57BL/6J mice were obtained from the Chinese Academy of Medical Science’s Center of Medical Experimental Animals. NCG-*c-Kit*-Cas9-TM (NCG-X) mice were purchased from Gempharmatech. tdTomato-transgenic mice were purchased from Shanghai Model Organisms Center. *Ahr*^–/–^C57BL/6J mice were gifted by J. Yan (Third Military Medical University). The experiments used 6- to 8-week-old mice, except for NCG-X mice, which were 4 to 5 weeks old. Animals were maintained in pathogen-free environments at 20–21 °C with 60–70% relative humidity on a 12-h light/12-h dark cycle in the Chinese Academy of Medical Science’s animal facilities. The Chinese Academy of Medical Science’s Animal Care and Use Committee gave its approval for all investigations that used mice.

For colorectal carcinoma models, WT, *Ahr*^–/–^ or tdTomato-transgenic mice were inoculated subcutaneously with 5 × 10^5^ MC38 cells into the right flank. Humanized mice were inoculated subcutaneously with 5 × 10^6^ HCT116 cells into the right flank. For breast carcinoma models, WT, *Ahr*^–/–^ or tdTomato-transgenic mice were implanted with 5 × 10^5^ E0771 cells into the inguinal mammary gland (sample size of *n* = 6 or 10).

### Human information and samples

Peripheral blood samples were obtained from the First Affiliated Hospital of Zhengzhou University. UCB samples were obtained from Women and Children’s Hospital, Xiamen University. Human BM samples were obtained from Peking University People’s Hospital. Ethical permission was granted by the Clinical Trial Ethics Committee of First Affiliated Hospital of Zhengzhou University (2019-KY-256), Women and Children’s Hospital, Xiamen University (KY-2019-073) and Peking University People’s Hospital (NKRDP2021005-EC-2). Every participant signed a written informed consent form for their participation in the trial. Detailed information is presented in Supplementary Tables 1–6.

### Cell preparation and in vitro treatment

For mouse MEPs, cells were isolated from BM by flow cytometry. Except for the c.f.u. assay, cells were cultured in StemSpan SFEM II (STEMCELL Technologies) with 50 μM β-mercaptoethanol and mouse cytokines (2.5 U ml^−1^ EPO, 10 ng ml^−1^ interleukin-3 (IL-3), 10 ng ml^−1^ IL-6, 25 ng ml^−1^ stem cell factor (SCF), 50 ng ml^−1^ TPO, 10 ng ml^−1^ IL-11 and 25 ng ml^−1^ FLT3L). Human MEPs were isolated from UCB or BM by flow cytometry. Except for c.f.u. assays, human MEPs were cultured in StemSpan SFEM II containing human cytokines (2.5 U ml^−1^ EPO, 10 ng ml^−1^ IL-3, 10 ng ml^−1^ IL-6, 100 ng ml^−1^ SCF, 25 ng ml^−1^ TPO, 10 ng ml^−1^ IL-11 and 100 ng ml^−1^ FLT3L). Mouse MEPs were treated with PBS or Kyn (5 μM), Kyn or Kyn combined with Ro5-3335 (2 μM) or SR1 (1 μM). Human MEPs were treated with PBS or Kyn (5 μM). After the indicated amounts of time, mouse or human MEPs were collected for analysis. Detailed reagent information is available in Supplementary Table 7.

### Animal in vivo treatment

C57BL/6J mice were inoculated with MC38 or E0771 cells, and RBCs and PLTs were counted once a week with a Sysmex XN-1000V until tumors were approximately 2,000 mm^3^. Different doses of Kyn (100 to 400 μg) were injected (i.p.) into C57BL/6J mice, and serum Kyn levels were detected at 1, 3, 8, 12 and 24 h by HPLC to obtain time–concentration curves. C57BL/6J mice were injected with Kyn (i.p.; 400 μg every 12 h) for 14 d, and blood samples were taken for RBC and PLT counting. BM cells were used for EryP and MkP analysis, and MEPs were isolated and stained for RUNX1. MC38 or E0771 tumor-bearing mice were treated with PBS or Kyn, Kyn or Kyn combined with SR1 (i.p.; 50 μg every day), PBS or SR1 or PBS or 1-MT (5 mg ml^−1^ in drinking water, 3–4 ml per mouse per day) for 2 weeks, and the numbers of RBCs, PLTs, EryPs and MkPs were quantified. MEPs were isolated for AhR, SLC7A8 or RUNX1 staining.

For the in vivo study of humanized mice, human CD34^+^ cells were isolated from human UCB with a CD34 microbeads kit (Miltenyi Biotec). NCG-X mice were transferred (intravenously) with 5 × 10^4^ CD34^+^ cells, and HCT116 cells were inoculated subcutaneously in these mice after 14 weeks. Concurrently, mice were injected (i.p.) with recombinant human EPO (30 IU per mouse), recombinant human TPO (250 ng per mouse) and recombinant human IL-3 (25 ng per mouse) every 3 d to promote RBC and PLT production. Two weeks later, mice were treated with chlorophosphate liposome (i.p.; 200 μl per mouse) every 2 d three times. Peripheral human-derived RBCs and PLTs of the treated mice were detected by flow cytometry at week 18. For Kyn and SR1 treatment, humanized NCG-X mice were treated with Kyn or Kyn combined with SR1, as previously described, after CD34^+^ cells were transferred at 14 weeks. Similarly, recombinant human EPO, recombinant human TPO, recombinant human IL-3 and chlorophosphate liposomes were administrated for the indicated lengths of time, and peripheral human-derived RBCs and PLTs were detected by flow cytometry.

For BM transplantation, tdTomato-transgenic mice were exposed to a split dose of 9 Gy by X-ray with 4-h intervals. Fresh BM cells (1 × 10^6^) from WT or *Ahr*^–/–^ mice were transplanted (intravenously) into each irradiated recipient. After 6 weeks, these recipient mice were injected with MC38 or E0771 cells. All donor-derived tdTomato^–^ RBCs or PLTs were further counted by flow cytometry at the indicated times after MC38 or E0771 inoculation.

### Flow cytometry and cell sorting

Single-cell suspensions from mouse BM or human UCB or BM were prepared. All samples were stained with a Zombie Aqua Fixable Viability kit (Biolegend) for 15 min at room temperature before they were stained with antibodies. All antibodies were diluted 1:100 with cell sorting buffer. An Invitrogen Attune NxT flow cytometer and Invitrogen Attune NxT software were used for data collection. Cell sorting was performed with a Sony MA900 flow cytometer. Data were analyzed with FlowJo software. For detailed antibody information, please see Supplementary Table 8.

### Digital western blotting

MEPs isolated from C57BL/6J mice were treated with PBS, Kyn or Kyn combined with SR1 for 48 h, and MEPs isolated from *Ahr*^–/–^ mice were treated with PBS or Kyn for 48 h. Equal numbers of MEPs from each group were collected, lysed in M2 lysis buffer and sonicated. Proteins were probed with different antibodies (Supplementary Table 8) by using a Digital Western Blot system (Jess, Bio-Techne). Digital western blot data were captured using Compass for SW.

### Immunofluorescence

Cells were fixed in 4% paraformaldehyde and permeabilized with 0.2% Triton X-100 on slides. Fixed cells were blocked in 5% bovine serum albumin and incubated with different antibodies (1:200 dilution; Supplementary Table 8) at 4 °C overnight, and cells were then washed and incubated with secondary antibodies (1:500 dilution) for 1 h at room temperature. Slides were counterstained with DAPI. Images were collected on a Nikon A1 HD25 microscope using NIS-Elements AR software. Immunofluorescence intensity was analyzed with ImageJ v1.52 software. Mean fluorescence intensity within the AhR^+^, RUNX1^+^ or SLC7A8^+^ region of interest was calculated from 15 random cells per slide.

### Transfection of siRNAs into mouse MEPs

Mouse MEPs were resuspended in 20 μl of cell-specific nuclear transfection solution (P3 Primary Cell kit, Lonza) with 50 nM *Slc1a5*, *Slc7a5*, *Slc7a8* or *Pat4* siRNA and transfected with a Lonza 4D X-Unit with the DK-100 program. After transfection, cells were cultured with the appropriate aforementioned medium and cytokines. siRNA sequences are provided in Supplementary Table 9.

### Stable transfection of shRNAs

Human MEPs were infected with lentivirus for stable transfection. Lentiviruses containing pLKO.1-*AhR*-SH green fluorescent protein (GFP) or scrambled control were packaged in HEK 293T cells with psPAX2 (Addgene, 12260) and pMD2.G packaging plasmid (Addgene, 12259). Human MEPs were then infected by adding a ratio of 1:2 (vol:vol) viral supernatant:fresh medium containing 6 μg ml^−1^ polybrene (Sigma) and were centrifuged at 1,200*g* for 60 min at 32 °C. Four hours after spinning inoculation, the supernatant was replaced with fresh medium and cultured for another 20 h. Cells with GFP fluorescence were then isolated by flow cytometry cell sorting. shRNA sequences are provided in Supplementary Table 9.

### Luciferase assays

One hundred nanograms of *Renilla* luciferase plasmid pRL-SV40, 1 μg of firefly luciferase plasmid pGL4.10-*Runx1* P1 promoter-luciferase or pGL4.10-*Slc7a8* promoter-luciferase and 1 μg of pCMV6-*Ahr* plasmid were transfected into NIH-3T3 cells for 12 h. Transfected cells were then treated with 100 μM Kyn for 48 h. One hundred nanograms of *Renilla* luciferase plasmid pRL-SV40, 1 μg of firefly luciferase plasmid pGL4.10-*Itgb2b* promoter-luciferase, pGL4.10-*Gp1ba* promoter-luciferase or pGL4.10-*Klf1* promoter-luciferase and 1 μg of pCMV6-*Runx1* plasmid were transfected into NIH-3T3 cells for 12 h. Transfected cells were then treated with 100 μM Kyn for 48 h. One hundred nanograms of *Renilla* luciferase plasmid pRL-SV40, 1 μg of firefly luciferase plasmid pGL4.10-*RUNX1* promoter-luciferase or pGL4.10-*SLC7A8* promoter-luciferase and 1 μg of pCMV6-*AhR* plasmid were transfected into HEK 293T cells for 12 h, and transfected cells were treated with 100 μM Kyn for 48 h. Cell lysis was performed with a Dual Luciferase Reporter Assay (Promega), and the ratio of firefly luciferase activity to *Renilla* luciferase activity was analyzed and calculated using GloMax Multi Plus (Promega).

### RT–qPCR

TRIzol (Invitrogen) was used to extract total RNA from cells. A high-capacity cDNA reverse transcription kit (Applied Biosystems) was used to transcribe total RNA into cDNA. The primer sequences are shown in Supplementary Table 9. An ABI QuantStudio 3 (Applied Biosystems) and QuantStudio Design & Analysis were used for RT–qPCR. Values are shown as mean ± s.d. from three independent experiments.

### CUT&RUN-qPCR assay

CUT&RUN-qPCR was performed by using a CUT&RUN assay kit (Cell Signaling Technology) according to the manufacturer’s protocol. Briefly, mouse MEPs were treated with Kyn for 48 or 72 h. Cells were permeabilized with digitonin, and antibody to AhR or RUNX1 and a Protein A-Protein G-Micrococcal Nuclease (pAG-MNase) were used to isolate specific protein–DNA complexes. DNA fragments were collected using DNA spin columns (Zymo Research), and enriched DNA was identified and quantified using qPCR. The primer sequences are shown in Supplementary Table 9.

### HPLC analysis of serum Kyn levels

Serum Kyn determination was performed by Dionex UltiMate3000. Mouse serum was mixed with 10% trichloroacetic acid (1:1) for 30 min, and the mixture was centrifuged at 14,000*g* for 10 min at 4 °C. Then, 20 µl of supernatant was injected into the HPLC instrument for quantification. The mobile phase consisted of 15 mM sodium acetate containing 8.0% acetonitrile (pH 4.0), which was pumped at a flow rate of 1.0 ml min^−1^. The analytical column was a C18 chromatographic column (Thermo Fisher Scientific, 250 mm × 4.6 mm, 5 μm) and kept at 30 °C. Kyn was measured at a wavelength of 360 nm.

### LC–MS analysis of MEP intracellular Kyn level

Intracellular Kyn determination was performed with an Agilent 6495. Mouse MEPs were collected and washed twice in PBS and lysed with 50 μl of 80% (vol/vol) methanol/water and centrifuged at 15,000*g* for 15 min at 4 °C. The supernatant was collected for direct detection or was stored at −80 °C. An Agilent 6495 triple quadrupole LC–MS system and C18 chromatographic column (Thermo Fisher Scientific, 100 mm × 2.1 mm, 1.7 μm) were used. For LC separation, gradient elution was performed using 0.1% formic acid as solvent A and pure acetonitrile as solvent B. The flow rate was 0.3 ml min^−1^, and the injection volume was 10 μl. The following gradient program was used: 0–1 min, 95% A; 1–6 min, 95% A to 5% A; 6–7 min, 5% A; 7–7.2 min, 5% A to 95% A and 7.2–11 min, 95% A. The total run time was 11 min for each sample, and the data were collected by Data Acquisition.

### C.f.u. assays

Mouse MEPs were cultured in collagen-based medium with β-mercaptoethanol and mouse cytokines EPO, TPO, SCF, IL-3, IL-6 and IL-11, as described above. Cells were cultured with the addition of 2 μM Ro5-3335 (MedChemExpress), 100 μM BCH (MedChemExpress), 5 μM Kyn or PBS for 7 d. Human MEPs were cultured in collagen-based medium with recombinant human EPO, TPO, SCF, IL-3, IL-6 and IL-11, as described above. Cells were cultured with the addition of 5 μM Kyn or PBS for 12 d. A double-chamber slide kit (STEMCELL Technologies) was used for dual megakaryocyte/erythroid c.f.u. assays. Collagen-based medium in double-chamber slides was dehydrated and fixed according to the manufacturer’s protocol. All antibody incubations were performed for 30 min at room temperature. Cultures were rehydrated in Tris/NaCl buffer containing 10% goat and 2.5% mouse serum. For mouse and human c.f.u. megakaryocyte staining, we used rabbit anti-CD41 (1:100 dilution) and then goat anti-rabbit IgG (H + L) cross-adsorbed secondary antibody AF488 (1:500 dilution). For mouse and human c.f.u. erythroid staining, we used mouse anti-CD71 (1:100 dilution) and then donkey anti-mouse IgG (H + L) highly cross-adsorbed secondary antibody AF594 (1:500 dilution). Colonies were scored based on CD41 and CD71 staining as megakaryocyte only, erythroid only or megakaryocyte plus erythroid. The c.f.u. values were observed with a Leica DMi8 by using LAS X Life Science Software.

### Quantification and statistical analysis

All experiments were performed with at least three biological repeats. Results are expressed as mean ± s.d. or mean ± s.e.m., as indicated, and were analyzed by one-way ANOVA followed by a Bonferroni post hoc test or by a two-tailed Student’s *t*-test. A *P* value of <0.05 was considered statistically significant. Analyses were conducted using GraphPad 8.0 software.

### Reporting summary

Further information on research design is available in the Nature Portfolio Reporting Summary linked to this article.

## Online content

Any methods, additional references, Nature Portfolio reporting summaries, source data, extended data, supplementary information, acknowledgements, peer review information; details of author contributions and competing interests; and statements of data and code availability are available at 10.1038/s41590-023-01662-3.

### Supplementary information

Reporting Summary

Supplementary Tables 1–9 Supplementary Table 1. Clinical information of healthy individuals. Supplementary Table 2. Clinical information of individuals with colon cancer. Supplementary Table 3. Clinical information of individuals with lung cancer. Supplementary Table 4. Clinical information of individuals with breast cancer. Supplementary Table 5. Clinical information of individuals with leukemia. Supplementary Table 6. Clinical information of BM donors and individuals with lymphoma. Supplementary Table 7. In vitro and in vivo treatment reagents. Supplementary Table 8. Antibodies for flow cytometry, digital western blotting, immunofluorescence and CUT&RUN. Supplementary Table 9. Sequences of siRNA, shRNA and PCR primers

### Source data

Source Data Fig. 1 Statistical source data.

Source Data Fig. 1 Unprocessed c.f.u. image.

Source Data Fig. 2 Statistical source data.

Source Data Fig. 2 Unprocessed fluorescence image.

Source Data Fig. 3 Statistical source data.

Source Data Fig. 3 Unprocessed western blot.

Source Data Fig. 4 Statistical source data.

Source Data Fig. 4 Unprocessed fluorescence image.

Source Data Fig. 5 Statistical source data.

Source Data Fig. 5 Unprocessed fluorescence image.

Source Data Fig. 6 Statistical source data.

Source Data Fig. 6 Unprocessed c.f.u. and fluorescence image.

Source Data Fig. 7 Statistical source data.

Source Data Fig. 7 Unprocessed fluorescence image.

Source Data Extended Data Fig. 1 Statistical source data.

Source Data Extended Data Fig. 2 Statistical source data.

Source Data Extended Data Fig. 2 Unprocessed fluorescence image.

Source Data Extended Data Fig. 3 Statistical source data.

Source Data Extended Data Fig. 3 Unprocessed fluorescence image.

Source Data Extended Data Fig. 4 Statistical source data.

Source Data Extended Data Fig. 4 Unprocessed fluorescence image.

Source Data Extended Data Fig. 5 Statistical source data.

Source Data Extended Data Fig. 5 Unprocessed fluorescence image.

Source Data Extended Data Fig. 6 Statistical source data.

Source Data Extended Data Fig. 6 Unprocessed fluorescence image.

Source Data Extended Data Fig. 7 Statistical source data.

Source Data Extended Data Fig. 7 Unprocessed fluorescence image.

## Data Availability

The main data supporting the results in this study are available within the paper and its Supplementary Information. Source data are provided with this paper.
