# Publisher Correction: Tumor cell-released kynurenine biases MEP differentiation into megakaryocytes in individuals with cancer by activating AhR–RUNX1

**DOI:** 10.1038/s41590-023-01699-4

**Published:** 2023-11-07

**Authors:** Li Zhou, Dongxiao Wu, Yabo Zhou, Dianheng Wang, Haixia Fu, Qiusha Huang, Guohui Qin, Jie Chen, Jiadi Lv, Shaoyang Lai, Huafeng Zhang, Ke Tang, Jingwei Ma, Roland Fiskesund, Yi Zhang, Xiaohui Zhang, Bo Huang

**Affiliations:** 1 Department of Immunology & National Key Laboratory of Medical Molecular Biology, Institute of Basic Medical Sciences, Chinese Academy of Medical Sciences (CAMS) & Peking Union Medical College, Beijing, China; 2 Peking University People’s Hospital, Peking University Institute of Hematology, National Clinical Research Center for Hematologic Disease, Beijing Key Laboratory of Hematopoietic Stem Cell Transplantation, Beijing, China; 3 Biotherapy Center and Cancer Center, The First Affiliated Hospital of Zhengzhou University, Zhengzhou, China; 4 The Department of Obstetrics, Women and Children’s Hospital, School of Medicine, Xiamen University, Xiamen, China; 5 Department of Pathology, School of Basic Medicine, Tongji Medical College, Huazhong University of Science and Technology, Wuhan, China; 6 Department of Biochemistry and Molecular Biology, Tongji Medical College, Huazhong University of Science and Technology, Wuhan, China; 7 Department of Immunology, School of Basic Medicine, Tongji Medical College, Huazhong University of Science and Technology, Wuhan, China; 8 Department of Clinical Immunology and Transfusion Medicine, Karolinska University Hospital, Stockholm, Sweden; 9 Department of Medicine, Karolinska Institutet, Huddinge, Sweden

**Keywords:** Haematopoietic stem cells, Haematopoietic stem cells

Correction to: *Nature Immunology* 10.1038/s41590-023-01662-3, published online 2 November 2023.

In the version of the article initially published, there were errors in Fig. 1c and 2d where “LSK” mistakenly appeared as “LK” and “Percentage in LK” appeared as “LK (%)”. These errors have been corrected in the HTML and PDF versions of the article.

