# Supplementary material for: Tumor cell-released kynurenine biases MEP differentiation into megakaryocytes in individuals with cancer by activating AhR–RUNX1
Source: Nat Immunol. 2023 Nov 2;24(12):2042–52. doi: 10.1038/s41590-023-01662-3 (PMC10681900; doi:10.1038/s41590-023-01662-3)
Supplement: Supplementary file 1 — Reporting Summary [file 41590_2023_1662_MOESM1_ESM.pdf]

Corresponding author(s): Yi Zhang, Xiaohui Zhang, Bo Huang

Last updated by author(s): Sep 4, 2023

Reporting Summary

Nature Portfolio wishes to improve the reproducibility of the work that we publish. This form provides structure for consistency and transparency in reporting. For further information on Nature Portfolio policies, see our Editorial Policies and the Editorial Policy Checklist.

Statistics

For all statistical analyses, confirm that the following items are present in the figure legend, table legend, main text, or Methods section.

n/a	Confirmed	
☐	☒	The exact sample size (*n*) for each experimental group/condition, given as a discrete number and unit of measurement
☐	☒	A statement on whether measurements were taken from distinct samples or whether the same sample was measured repeatedly
☐	☒	The statistical test(s) used AND whether they are one- or two-sided<br>*Only common tests should be described solely by name; describe more complex techniques in the Methods section.*
☒	☐	A description of all covariates tested
☒	☐	A description of any assumptions or corrections, such as tests of normality and adjustment for multiple comparisons
☐	☒	A full description of the statistical parameters including central tendency (e.g. means) or other basic estimates (e.g. regression coefficient) AND variation (e.g. standard deviation) or associated estimates of uncertainty (e.g. confidence intervals)
☐	☒	For null hypothesis testing, the test statistic (e.g. *F*, *t*, *r*) with confidence intervals, effect sizes, degrees of freedom and *P* value noted<br>*Give P values as exact values whenever suitable.*
☒	☐	For Bayesian analysis, information on the choice of priors and Markov chain Monte Carlo settings
☒	☐	For hierarchical and complex designs, identification of the appropriate level for tests and full reporting of outcomes
☒	☐	Estimates of effect sizes (e.g. Cohen's *d*, Pearson's *r*), indicating how they were calculated

Our web collection on statistics for biologists contains articles on many of the points above.

Software and code

Policy information about availability of computer code

Data collection	Fow cytometric datas were captured using Invitrogen Attune NxT software v 3.1.2. qPCR data were captured using QuantStudio Design & Analysis Software v1.5.2. HPLC-MS data were captured using Data Acquisition v10.1. Fluorescence data were captured using NIS-Elements AR 5.20.00 and LAS X Life Science Software, v3.7.3.23245. Digital western blot data were captured using Compass for SW v6.1.0.
Data analysis	GraphPad Prism v8.0 was used for analysis of in vivo and in vitro phenotype assays and for graph production. FlowJo v10.5.3 was used for analysis of flow cytometry data. Image J v1.52 were used for visualization and presentation of fluorescence imaging. For further details on these Methods and specific references please see Methods section.

For manuscripts utilizing custom algorithms or software that are central to the research but not yet described in published literature, software must be made available to editors and reviewers. We strongly encourage code deposition in a community repository (e.g. GitHub). See the Nature Portfolio guidelines for submitting code & software for further information.

Data

Policy information about availability of data

All manuscripts must include a data availability statement. This statement should provide the following information, where applicable:

- Accession codes, unique identifiers, or web links for publicly available datasets
- A description of any restrictions on data availability
- For clinical datasets or third party data, please ensure that the statement adheres to our policy

The main data supporting the results in this study are available within the paper and its Supplementary Information. The raw data analyzed for this study are provided as source data.

Research involving human participants, their data, or biological material

Policy information about studies with human participants or human data. See also policy information about sex, gender (identity/presentation), and sexual orientation and race, ethnicity and racism.

Reporting on sex and gender	The findings in this study used both male and female samples except for breast cancer. Breast cancer patients were mostly women, so we only collected sample from female.
Reporting on race, ethnicity, or other socially relevant groupings	The findings in this study were not involved in race, ethnicity, or other socially relevant groupings.
Population characteristics	Blood samples were obtained from patients with colon, lung and breast cancer at stage III or IV. Bone marrow were collected from T-ALL and B-ALL leukemia patients who did not received treatment before or received hematopoietic stem cell transplantation (HSCT). Bone marrow were collected from lymphoma patients who were not with bone marrow infiltration. All subjects provided written informed consent, and all experiments were done in male and female subjects. Ages ranged from 19-77.
Recruitment	Colon, lung, breast cancer patients, lymphoma patients, leukemia patients and healthy donors was were recruited by the First Affiliated Hospital of Zhengzhou University, Peking University People's Hospital and Women and Children's Hospital of Xiamen University. All colon, lung, breast cancer patients, lymphoma patients did not received treatment before. Part of leukemia patients received hematopoietic stem cell transplantation, another did not received treatment before. The selection bias did not impact our results.
Ethics oversight	Human protocol was approved by the Ethics Committee of the First Affiliated Hospital of Zhengzhou University(2019-KY-256), Women and Children's Hospital of Xiamen University(KY-2019-073) and Peking University People's Hospital (NKRDP2021005-EC-2).

Note that full information on the approval of the study protocol must also be provided in the manuscript.

Field-specific reporting

Please select the one below that is the best fit for your research. If you are not sure, read the appropriate sections before making your selection.

☒ Life sciences ☐ Behavioural & social sciences ☐ Ecological, evolutionary & environmental sciences

For a reference copy of the document with all sections, see nature.com/documents/nr-reporting-summary-flat.pdf

Life sciences study design

All studies must disclose on these points even when the disclosure is negative.

Sample size	Sample sizes were chosen based on the previous experience of the investigators with similar experimental models. No statistical methods were used to predetermine sample sizes but our sample sizes are similar to those reported in previous publications including those from our group (cancer cell. 2018;33(3):480-94; Blood. 2019;134 (18):1547–57).
Data exclusions	No data were excluded from analysis.
Replication	Replicates were used in all experiments as noted in text, figure legends and methods. All experiments presented for which replication was attempted were successfully replicated.
Randomization	For in vitro experiments, MEPs were divided equally to each group, then treated with drug agents or transduced with siRNA or lentivirus. For in vivo experiments, mice were randomly assigned to each group.
Blinding	During the in vivo experiments, investigators were blinded to group assignments. The experimental conditions were not blinded in other in vitro experiments, since the comparisons were objective and quantitative.

Reporting for specific materials, systems and methods

We require information from authors about some types of materials, experimental systems and methods used in many studies. Here, indicate whether each material, system or method listed is relevant to your study. If you are not sure if a list item applies to your research, read the appropriate section before selecting a response.

Materials & experimental systems

n/a	Involved in the study
☐	☒ Antibodies
☐	☒ Eukaryotic cell lines
☒	☐ Palaeontology and archaeology
☐	☒ Animals and other organisms
☒	☐ Clinical data
☒	☐ Dual use research of concern
☒	☐ Plants

Methods

n/a	Involved in the study
☒	☐ ChIP-seq
☐	☒ Flow cytometry
☒	☐ MRI-based neuroimaging

Antibodies

Antibodies used

For flow cytometry analysis:
PE anti-mouse Lineage Cocktail (Biolegend, Cat#133303, clone 145-2C11 ; RB6-8C5 ; RA3-6B2 ; Ter-119 ; M1/70, 1:20)
PE anti-mouse CD127 (Biolegend, Cat#135009, clone A7R34, 1:100)
APC anti-mouse CD117 (Biolegend, Cat#135108, clone 2B8, 1:100)
PE/Cyanine7 anti-mouse Ly-6A/E (Biolegend, Cat#108114, clone D7, 1:100)
Brilliant Violet 421 anti-mouse CD16/32 (Biolegend, Cat#101332, clone 93, 1:100)
CD34 Monoclonal , FITC (Invitrogen, Cat#11-0341-82, clone RAM34, 1:100)
PE anti-mouse CD41 (Biolegend,Cat#133906, clone MWReg30, 1:100)
PerCP/Cyanine5.5 anti-mouse CD105 (Biolegend, Cat#120416, clone MJ7/18, 1:100)
PE/Dazzle 594 anti-mouse CD150 (Biolegend, Cat#115936, clone TC15-12F12.2, 1:100)
Brilliant Violet 421 anti-mouse CD135 (Biolegend, Cat#135313, clone A2F10, 1:100)
TER-119, APC (eBioscience, Cat#17-5921-82, clone TER-119)
APC anti-human Lineage Cocktail (CD3, CD14, CD19, CD20, CD56) (Biolegend, Cat#348703, clone UCHT1; HCD14; HIB19; 2H7; HCD56, 1:20)
PE anti-human CD34 Antibody (Biolegend, Cat#343506, clone 581, 1:100)
Brilliant Violet 421 anti-human CD38 (Biolegend, Cat#356618, clone HB7, 1:100)
Brilliant Violet 650 anti-human CD123 (Biolegend, Cat#306019, clone 6H6, 1:100)
PE/Cyanine7 anti-human CD45RA (Biolegend, Cat# 304126, clone HI100, 1:100)
PE anti-human CD235a (Biolegend, Cat#349106, clone HI264, 1:100)
FITC anti-human CD41 (Biolegend, Cat#303704, clone HIP8, 1:100)
APC anti-human CD41 (Biolegend, Cat#303710 clone HIP8, 1:100)
APC/Cyanine7 anti-human CD71 (Biolegend, Cat#334110, clone CY1G4, 1:100)
FITC anti-human CD105 (Biolegend, Cat#323204, clone 43A3, 1:100)
Biotin anti-human CD34 (Biolegend, Cat#343524, clone 581, 1:100)
PE anti-human CD62P (Biolegend, Cat#304906, clone AK4, 1:100)
FITC anti-human CD41/CD61 (Biolegend, Cat#362804, clone PAC-1, 1:100)
For digital western blot analysis:
GATA1 Monoclonal antibody (Proteintech, Cat#60011-1-Ig, clone 5E2A8, 0.2 µL/test)
GATA2 Polyclonal antibody (Proteintech, Cat#11103-1-AP, 0.2 µL/test)
RUNX1 Polyclonal antibody (Proteintech, Cat#25315-1-AP, 0.2 µL/test)
TAL1 Polyclonal antibody (Proteintech, Cat#55317-1-AP, 0.2 µL/test)
KLF1 Monoclonal antibody (Santa Cruze, Cat#sc-166238, clone F-8, 0.2 µL/test)
FLI1 Polyclonal antibody (Proteintech, Cat#11347-1-AP, 0.2 µL/test)
c-Myb Polyclonal antibody (Proteintech, Cat#17800-1-AP, 0.2 µL/test)
β-Actin Monoclonal antibody (Cell Signaling Technology, Cat#3700, clone 8H10D10, 0.2 µL/test)
For Immunofluorescence analysis:
RUNX1 Polyclonal antibody (Proteintech, Cat#25315-1-AP, 1:200)
SLC7A8 Monoclonal antibody (LS BIO, Cat#LS-B11782-100, clone 4A6, 1:200)
AhR Monoclonal antibody (Genetex, Cat#GTX22769, clone RPT9, 1:200)
For CFU assay:
CD41 Polyclonal antibody (Proteintech, Cat#24552-1-AP, 1:100)
CD71 Monoclonal antibody (Proteintech, Cat#66180-1-Ig, clone 3C11F11, 1:100)
For CUT&RUN analysis:
AhR Monoclonal antibody (Genetex, Cat#GTX22769, clone RPT9, 3µL/test)
RUNX1 Monoclonal antibody (Santa Cruze, Cat#sc-365644, clone A-2, 3µL/test)
Secondary antibodies:
HRP-conjugated Affinipure Goat Anti-Mouse IgG(H+L) (Proteintech, Cat#SA00001-1, 0.1 µL/test)
HRP-conjugated Affinipure Goat Anti-Rabbit IgG(H+L) (Proteintech, Cat# SA00001-2, 0.1 µL/test)
Donkey anti-Rabbit IgG (H+L) Highly Cross-Adsorbed Secondary Antibody, Alexa Fluor™ 488 (Invitrogen Cat#A-21206, 1:500)
Donkey anti-Mouse IgG (H+L) Highly Cross-Adsorbed Secondary Antibody, Alexa Fluor™ 488 (Invitrogen Cat#A-21202, 1:500)

	Donkey anti-Mouse IgG (H+L) Highly Cross-Adsorbed Secondary Antibody, Alexa Fluor™ 594 (Invitrogen Cat#A-21203, 1:500)<br>Donkey anti-Rabbit IgG (H+L) Highly Cross-Adsorbed Secondary Antibody, Alexa Fluor™ 594 (Invitrogen Cat#A-21207, 1:500)
Validation	For flow cytometry, western blot and immunofluorescence antibodies were validated as noted on manufacturer's website.<br>PE anti-mouse Lineage Cocktail https://www.biolegend.com/en-us/products/pe-anti-mouse-lineage-cocktail-with-isotype-ctrl-5804<br>PE anti-mouse CD127 https://www.biolegend.com/en-us/products/pe-anti-mouse-cd127-il-7ralpha-antibody-6190<br>APC anti-mouse CD117 https://www.biolegend.com/en-us/products/apc-anti-mouse-cd117-c-kit-antibody-6358<br>PE/Cyanine7 anti-mouse Ly-6A/E https://www.biolegend.com/en-us/products/pe-cyanine7-anti-mouse-ly-6a-e-sca-1-antibody-3137<br>Brilliant Violet 421 anti-mouse CD16/32 https://www.biolegend.com/en-us/products/brilliant-violet-421-anti-mouse-cd16-32-antibody-8598<br>CD34 Monoclonal , FITC https://www.thermofisher.cn/cn/zh/antibody/product/CD34-Antibody-clone-RAM34-Monoclonal/11-0341-82<br>PE anti-mouse CD41 https://www.biolegend.com/en-us/products/pe-anti-mouse-cd41-antibody-5897<br>PerCP/Cyanine5.5 anti-mouse CD105 https://www.biolegend.com/en-us/products/percp-cyanine5-5-anti-mouse-cd105-antibody-9225<br>PE/Dazzle 594 anti-mouse CD150 https://www.biolegend.com/en-us/products/pe-dazzle-594-anti-mouse-cd150-slam-antibody-12079<br>Brilliant Violet 421 anti-mouse CD135 https://www.biolegend.com/en-us/products/brilliant-violet-421-anti-mouse-cd135-antibody-8728<br>TER-119, APC https://www.thermofisher.cn/cn/zh/antibody/product/TER-119-Antibody-clone-TER-119-Monoclonal/17-5921-82<br>APC anti-human Lineage Cocktail https://www.biolegend.com/en-us/products/apc-anti-human-lineage-cocktail-cd3-cd14-cd19-cd20-cd56-8291<br>PE anti-human CD34 Antibody https://www.biolegend.com/en-us/products/pe-anti-human-cd34-antibody-6033<br>Brilliant Violet 421 anti-human CD38 https://www.biolegend.com/en-us/products/brilliant-violet-421-anti-human-cd38-antibody-12176<br>Brilliant Violet 650 anti-human CD123 https://www.biolegend.com/en-us/products/brilliant-violet-650-anti-human-cd123-antibody-7878<br>PE/Cyanine7 anti-human CD45RA https://www.biolegend.com/en-us/products/pe-cyanine7-anti-human-cd45ra-antibody-7055<br>PE anti-human CD235a https://www.biolegend.com/en-us/products/pe-anti-human-cd235a-glycophorin-a-antibody-6769<br>FITC anti-human CD41 https://www.biolegend.com/en-us/products/fitc-anti-human-cd41-antibody-736<br>APC anti-human CD41 https://www.biolegend.com/en-us/products/apc-anti-human-cd41-antibody-735<br>APC/Cyanine7 anti-human CD71 https://www.biolegend.com/en-us/products/apc-cyanine7-anti-human-cd71-antibody-9327<br>FITC anti-human CD105 https://www.biolegend.com/en-us/products/fitc-anti-human-cd105-antibody-3710<br>Biotin anti-human CD34 https://www.biolegend.com/en-us/products/biotin-anti-human-cd34-antibody-7097<br>PE anti-human CD62P https://www.biolegend.com/en-us/products/pe-anti-human-cd62p-p-selectin-antibody-595<br>FITC anti-human CD41/CD61 https://www.biolegend.com/en-us/products/fitc-anti-human-cd41-cd61-antibody-10179<br>GATA1 Monoclonal antibody https://www.ptglab.com/products/GATA1-Antibody-60011-1-Ig.htm<br>GATA2 Polyclonal antibody https://www.ptglab.com/products/GATA2-Antibody-11103-1-AP.htm<br>RUNX1 Polyclonal antibody https://www.ptglab.com/products/RUNX1-Antibody-25315-1-AP.htm<br>TAL1 Polyclonal antibody https://www.ptglab.com/products/TAL1-Antibody-55317-1-AP.htm<br>KLF1 Monoclonal antibody https://www.scbt.com/p/gklf-eklf-lklf-antibody-f-8?requestFrom=search<br>FLI1 Polyclonal antibody https://www.ptglab.com/products/FLI1-Antibody-11347-1-AP.htm<br>c-Myb Polyclonal antibody https://www.ptglab.com/products/MYB-Antibody-17800-1-AP.htm<br>β-Actin Monoclonal antibody https://www.cellsignal.com/products/primary-antibodies/b-actin-8h10d10-mouse-mab/3700<br>SLC7A8 Monoclonal antibody https://www.lsbio.com/antibodies/ihc-plus-slc7a8-antibody-lat2-antibody-clone-4a6-flow-if-immunofluorescence-ihc-wb-western-ls-b11782/346851<br>AhR Monoclonal antibody https://www.genetex.cn/Product/Detail/AHR-antibody-RPT9/GTX22769<br>CD41 Polyclonal antibody https://www.ptglab.com/products/ITGA2B-Antibody-24552-1-AP.htm<br>CD71 Monoclonal antibody https://www.ptglab.com/products/CD71-Antibody-66180-1-Ig.htm

Eukaryotic cell lines

Policy information about cell lines and Sex and Gender in Research

Cell line source(s)	Murine tumor cell lines E0771 (breast cancer) and MC38 (colon cancer); murine embryonic fibroblast cell line NIH3T3; human tumor cell lines HCT116 (colon cancer); human embryonic kidney cell line HEK 293T were purchased from the China Center for Type Culture Collection (Beijing, China).
Authentication	None of the cell lines were independently authenticated.
Mycoplasma contamination	Our cell lines are routinely tested for mycoplasma. None of the cell lines used in this study have tested positive for mycoplasma.
Commonly misidentified lines<br>(See ICLAC register)	None.

Animals and other research organisms

Policy information about studies involving animals; ARRIVE guidelines recommended for reporting animal research, and Sex and Gender in Research

Laboratory animals	6-8 weeks C57BL/6J mice were purchased from the Center of Medical Experimental Animals of the Chinese Academy of Medical Science (Beijing, China). 4-5 weeks NCG-c-Kit-Cas9-TM (NCG-X) mice were purchased from Gempharmatech (jiangsu, China). 6-8

	weeks tdTomato transgene mice were purchased from Shanghai Model Organisms Center (Shanghai, China). Ahr-/- mice were presented by Dr. Jun Yan (Third Military Medical University) and 6-8 weeks Ahr-/- mice were used for experiments.
Wild animals	No Wild animals were used in this study
Reporting on sex	For colon cancer model,we using both male and female mice; For breast cancer model,we using female mice.
Field-collected samples	No Field-collected samples were used in this study
Ethics oversight	These animals were maintained in the Animal Facilities of Chinese Academy of Medical Science under pathogen-free conditions. All studies involving mice were approved by the Animal Care and Use Committee of Chinese Academy of Medical Science.

Note that full information on the approval of the study protocol must also be provided in the manuscript.

Flow Cytometry

Plots

Confirm that:

☒ The axis labels state the marker and fluorochrome used (e.g. CD4-FITC).

☒ The axis scales are clearly visible. Include numbers along axes only for bottom left plot of group (a 'group' is an analysis of identical markers).

☒ All plots are contour plots with outliers or pseudocolor plots.

☒ A numerical value for number of cells or percentage (with statistics) is provided.

Methodology

Sample preparation	Preparation of single cell suspension from mouse bone marrow: bone marrow of hind limbs were harvested into a tissue culture dish with 5 mL PBS containing 0.5% FBS, then single-cell suspension were obtained by gently aspirating several times using the 1 mL syringe. Pass cell suspension through the cell strainer to eliminate clumps and debris, then centrifuge at 500 g for 5 minutes at 4°C to harvest the cell pellet, and repeat the wash step one time. Finally, re-suspend the cell pellet in PBS to the final concentration of 1 x 10^7 cells/mL and used for cell surface staining.<br>Isolation of PBMC from human bone marrow or umbilical cord blood: Dilute the blood at 1:1 with PBS, underlay the diluted blood with the same volume of Ficoll. Then centrifuge at 400 g for 20 minutes at room temperature.
Instrument	Invitrogen Attune NxT and Sony MA900
Software	FlowJo software
Cell population abundance	The single cell suspension from bone marrow or umbilical cord blood were staining with mouse or human MEP surface mark, then cells were sorted in Sony MA900 with purity of priority.
Gating strategy	For all experiments, debris was first excluded by a morphology gate based on FSC-A and SSC-A. Then, non-singlets were eliminated from analysis by a single cell gate based on FSC-H and FSC-A. All gates were set based on FMO (full-minus one) stains and isotype control antibodies after appropriate compensation using single-stained compensation controls.

☒ Tick this box to confirm that a figure exemplifying the gating strategy is provided in the Supplementary Information.
