# Supplementary figures and images for: Tumor cell-released kynurenine biases MEP differentiation into megakaryocytes in individuals with cancer by activating AhR–RUNX1
Source: Nat Immunol. 2023 Nov 2;24(12):2042–52. doi: 10.1038/s41590-023-01662-3 (PMC10681900; doi:10.1038/s41590-023-01662-3)

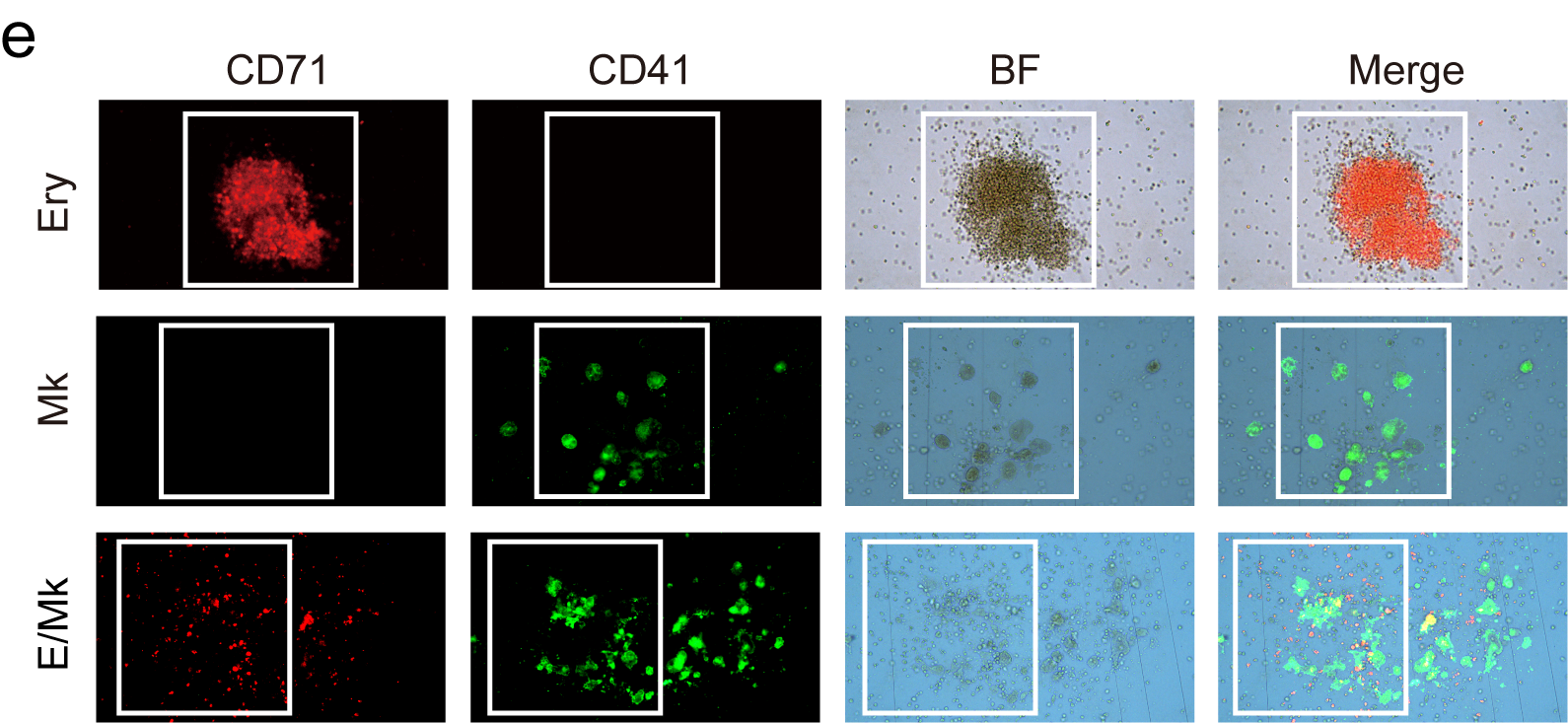

Supplement: Supplementary file 4 — Unprocessed c.f.u. image. [file 41590_2023_1662_MOESM4_ESM.tif]

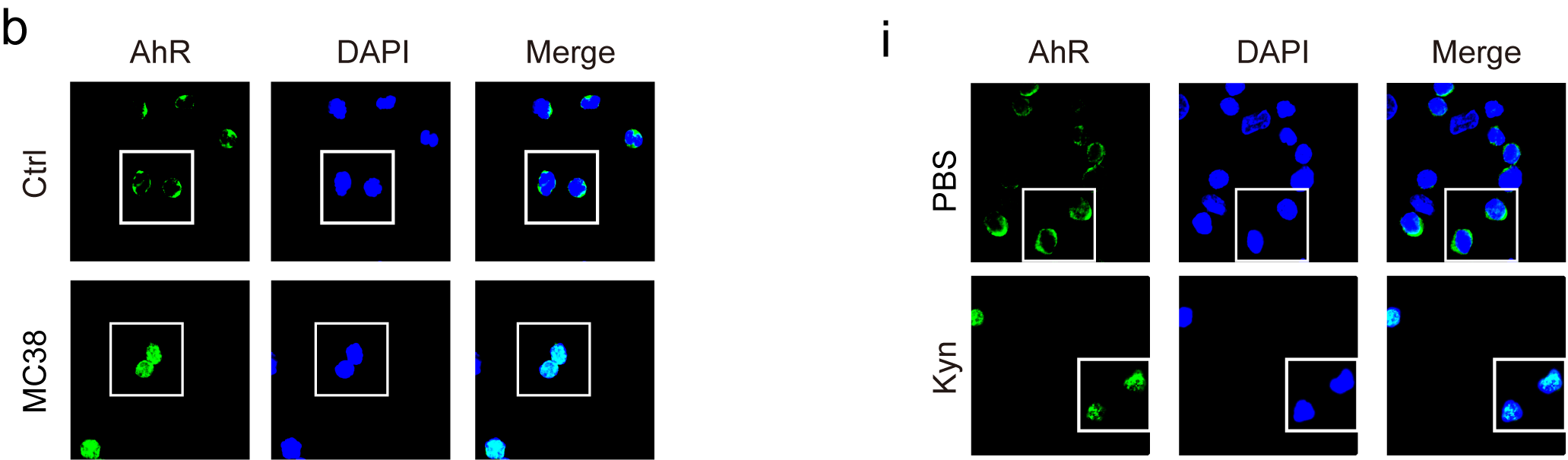

Supplement: Supplementary file 6 — Unprocessed fluorescence image. [file 41590_2023_1662_MOESM6_ESM.tif]

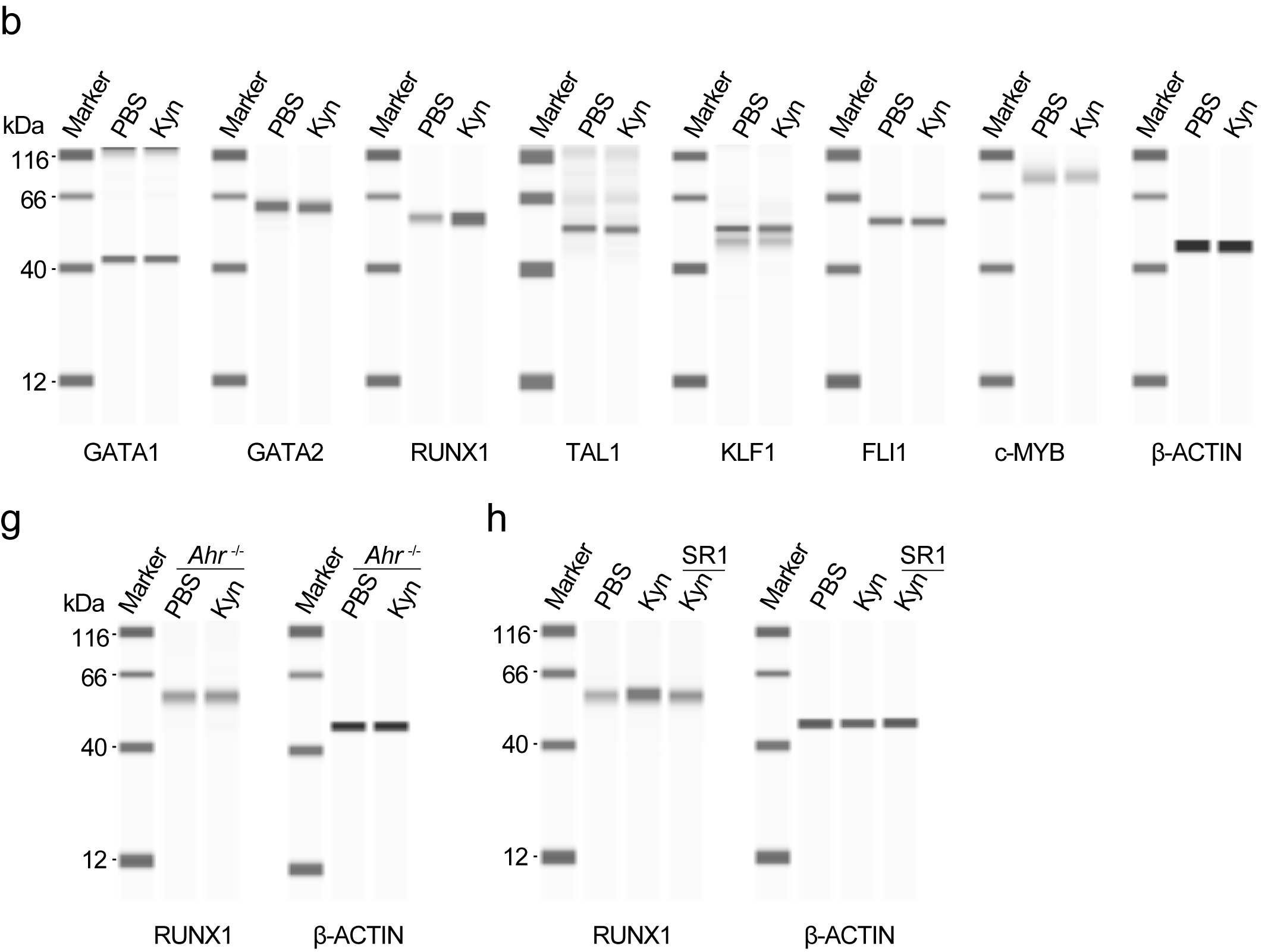

Supplement: Supplementary file 8 — Unprocessed western blot. [file 41590_2023_1662_MOESM8_ESM.tif]

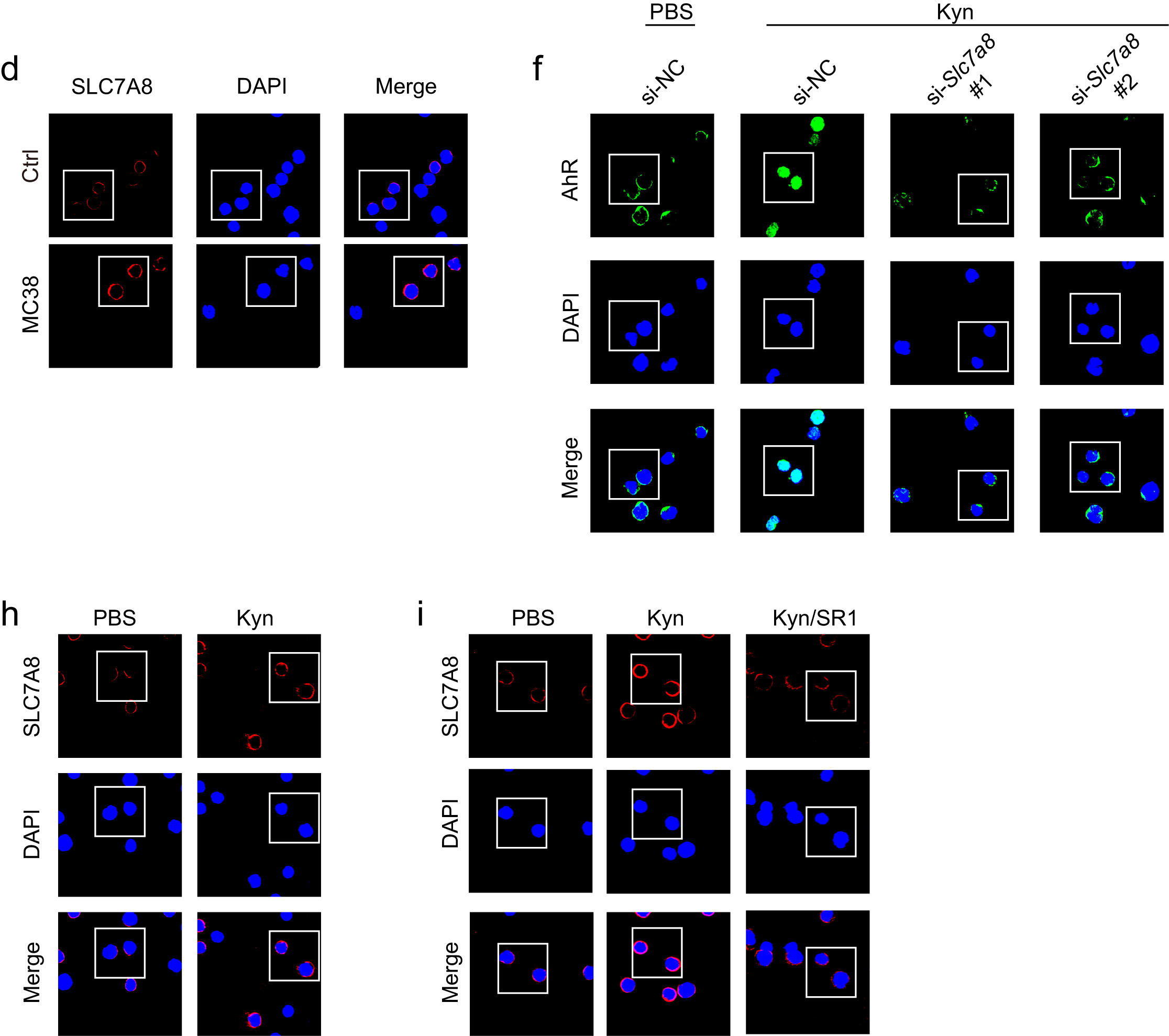

Supplement: Supplementary file 10 — Unprocessed fluorescence image. [file 41590_2023_1662_MOESM10_ESM.tif]

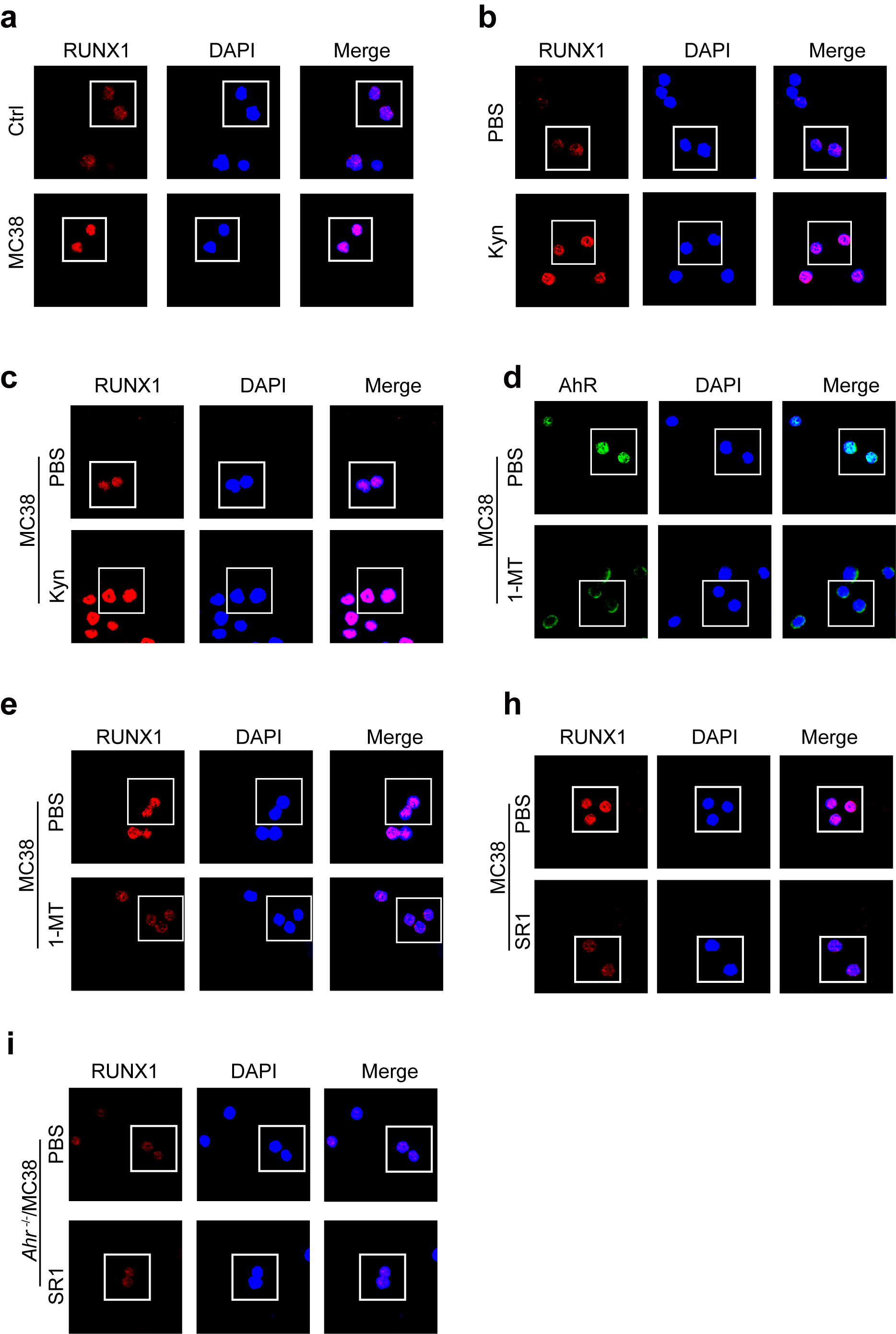

Supplement: Supplementary file 12 — Unprocessed fluorescence image. [file 41590_2023_1662_MOESM12_ESM.tif]

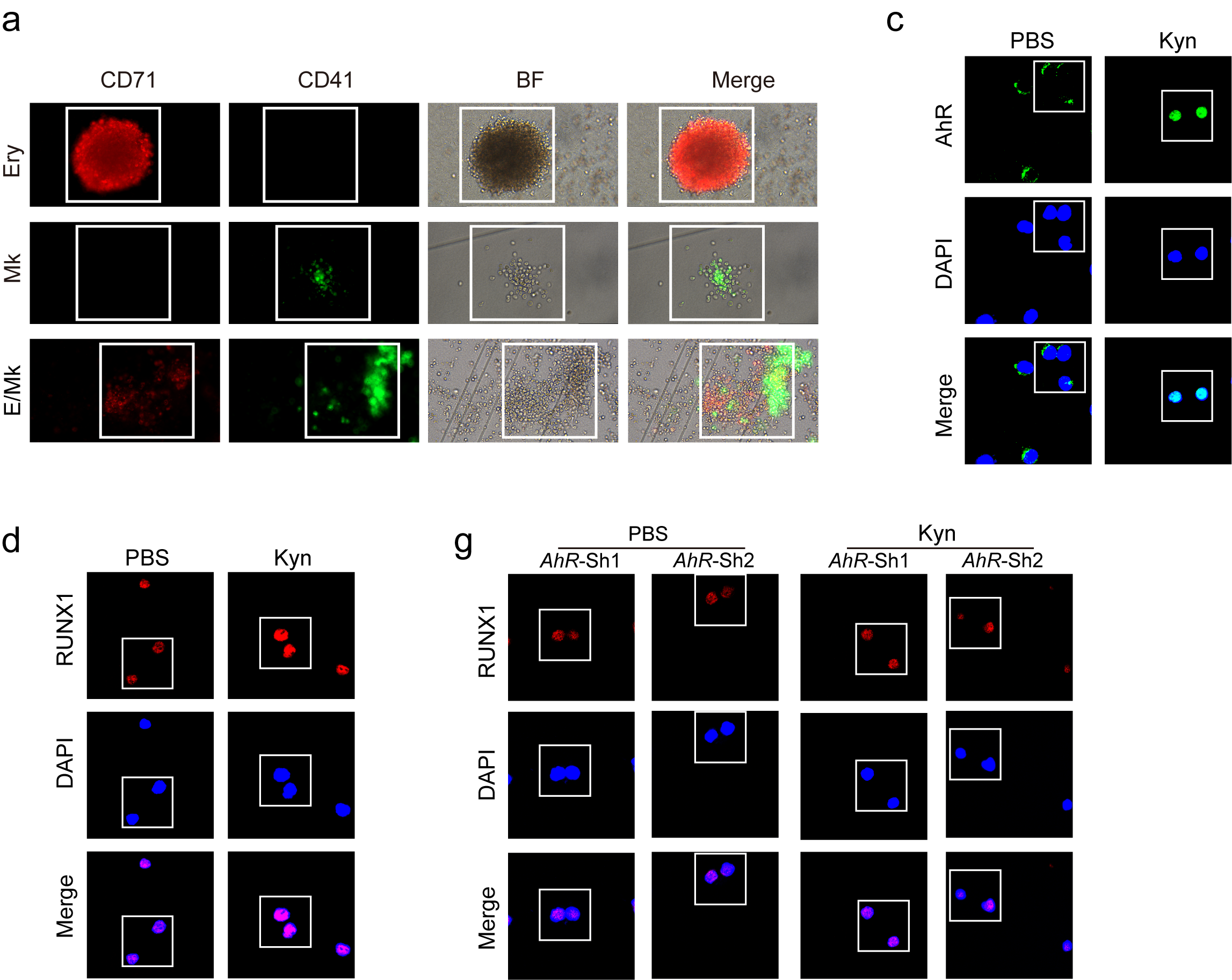

Supplement: Supplementary file 14 — Unprocessed c.f.u. and fluorescence image. [file 41590_2023_1662_MOESM14_ESM.tif]

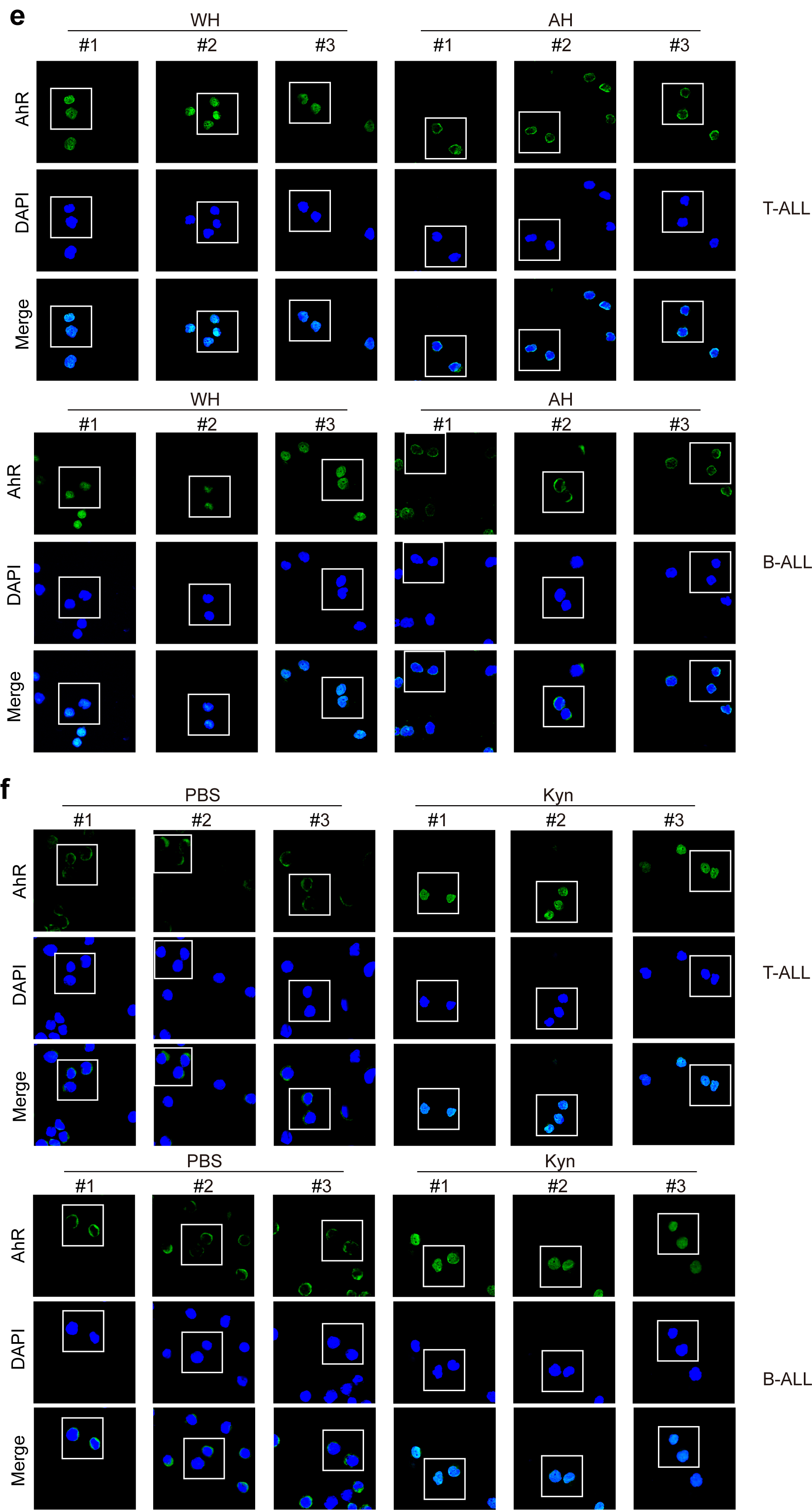

Supplement: Supplementary file 16 — Unprocessed fluorescence image. [file 41590_2023_1662_MOESM16_ESM.tif]

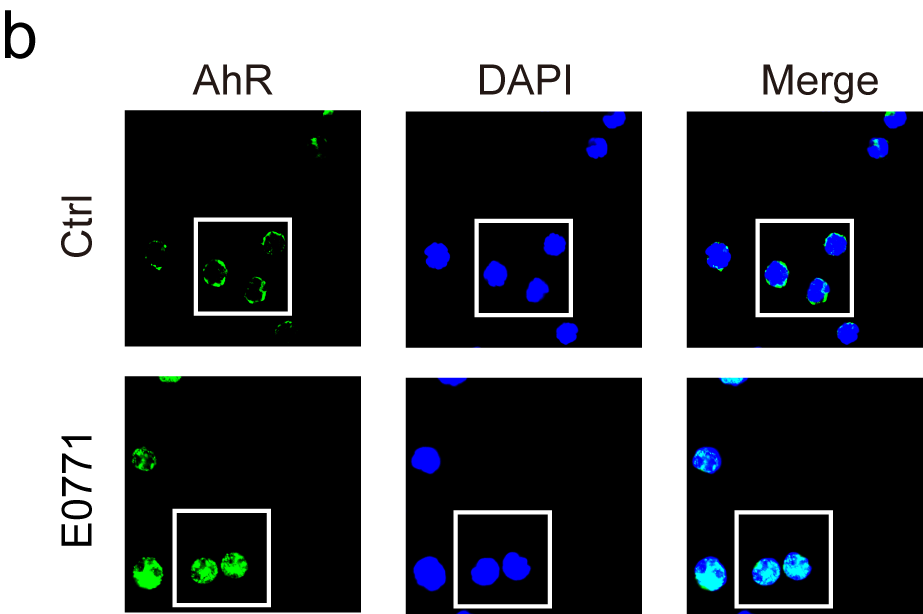

Supplement: Supplementary file 19 — Unprocessed fluorescence image. [file 41590_2023_1662_MOESM19_ESM.tif]

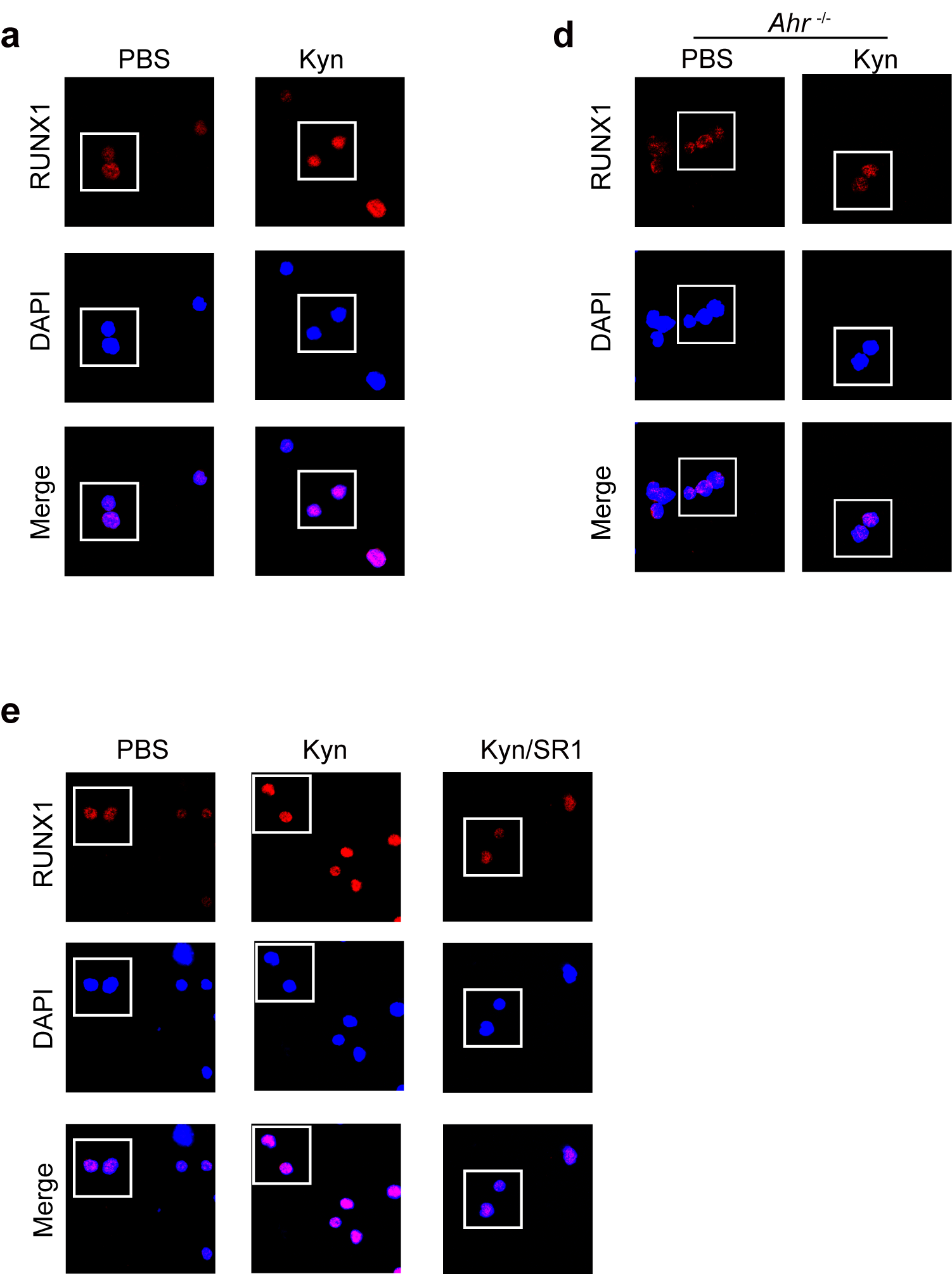

Supplement: Supplementary file 21 — Unprocessed fluorescence image. [file 41590_2023_1662_MOESM21_ESM.tif]

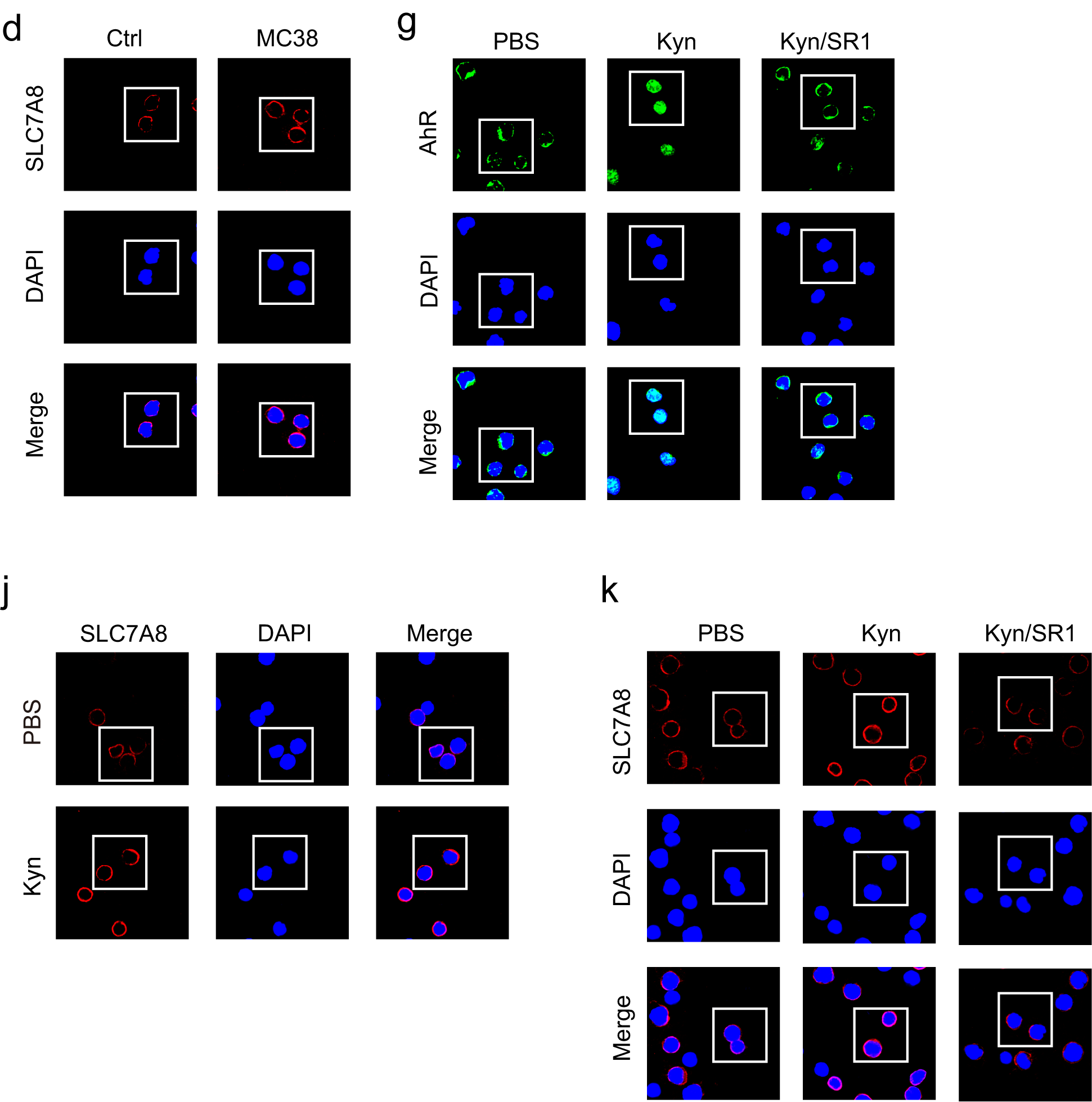

Supplement: Supplementary file 23 — Unprocessed fluorescence image. [file 41590_2023_1662_MOESM23_ESM.tif]

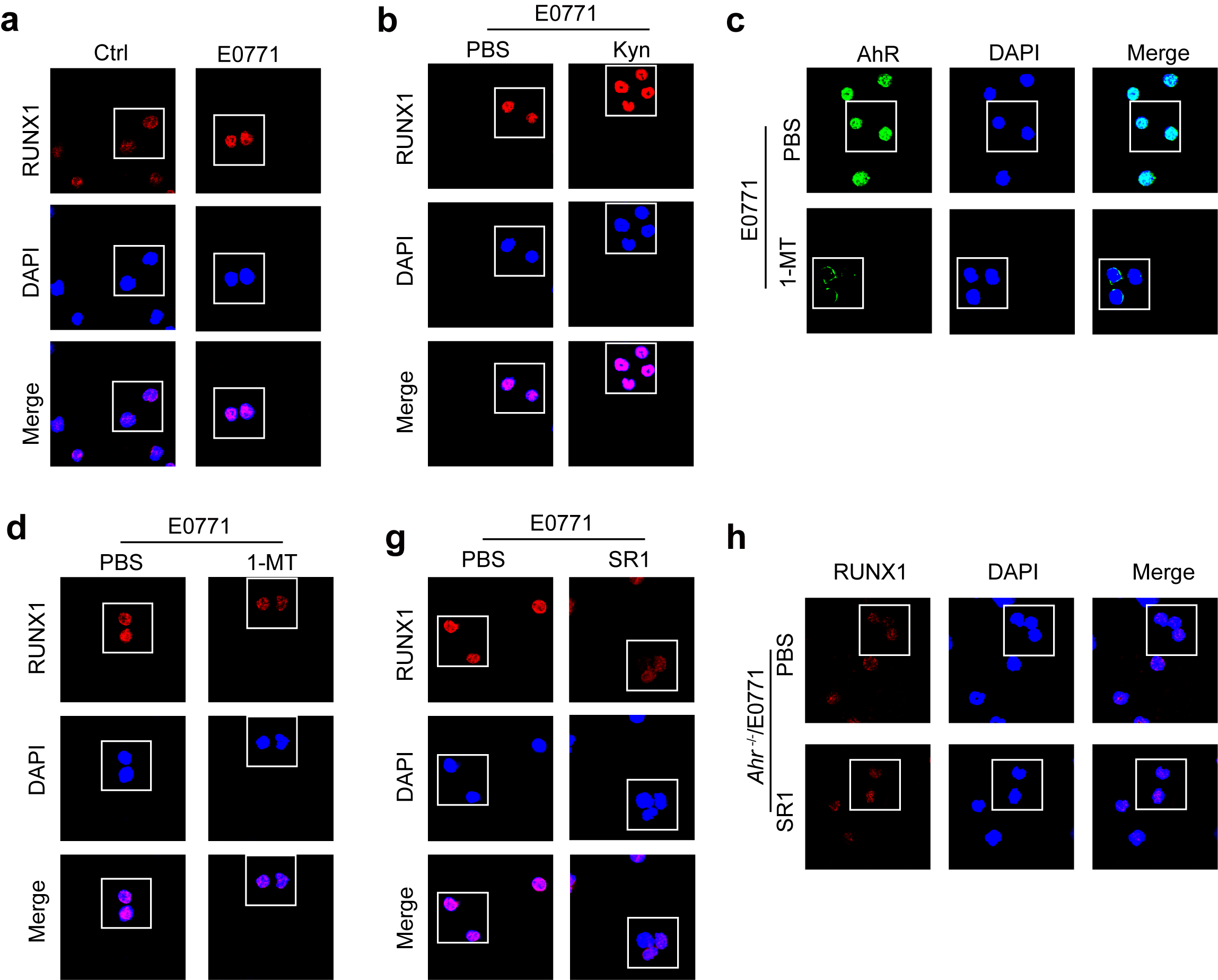

Supplement: Supplementary file 25 — Unprocessed fluorescence image. [file 41590_2023_1662_MOESM25_ESM.tif]

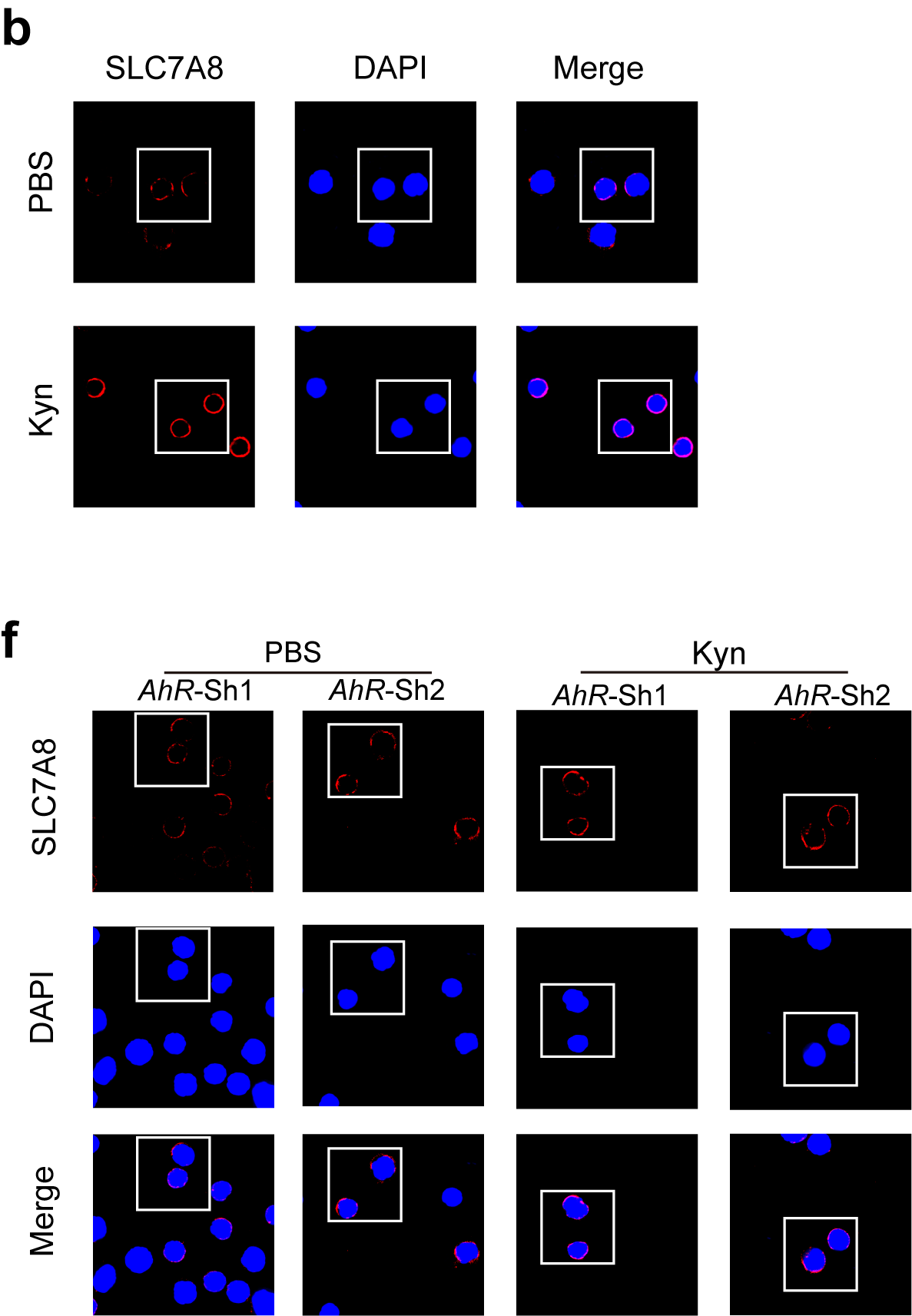

Supplement: Supplementary file 27 — Unprocessed fluorescence image. [file 41590_2023_1662_MOESM27_ESM.tif]

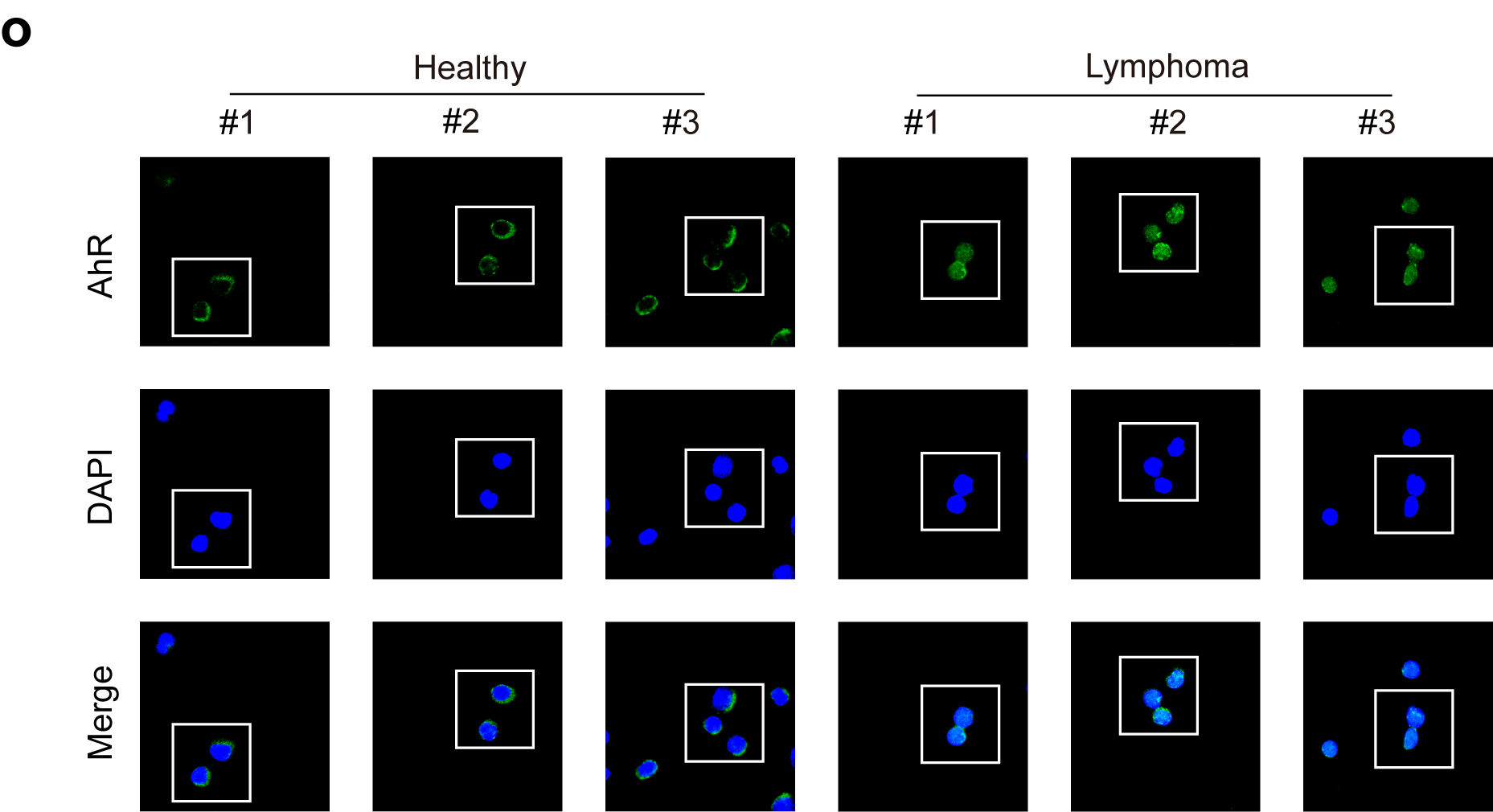

Supplement: Supplementary file 29 — Unprocessed fluorescence image. [file 41590_2023_1662_MOESM29_ESM.tif]
